# Plasmon-induced light concentration enhanced imaging visibility as observed by a composite-field microscopy imaging system†
†Electronic supplementary information (ESI) available: Experimental details including optical path refit details, synthesis and characterization of the nanoprobes, imaging operation and data analysis, Hep-2 cells-AgNPs scattering imaging details *etc.* See DOI: 10.1039/c6sc01055e


**DOI:** 10.1039/c6sc01055e

**Published:** 2016-04-25

**Authors:** Peng Fei Gao, Ming Xuan Gao, Hong Yan Zou, Rong Sheng Li, Jun Zhou, Jun Ma, Qiang Wang, Feng Liu, Na Li, Yuan Fang Li, Cheng Zhi Huang

**Affiliations:** a Key Laboratory of Luminescent and Real-Time Analytical Chemistry (Southwest University) , Ministry of Education , College of Pharmaceutical Sciences , Southwest University , Chongqing 400716 , China . Email: chengzhi@swu.edu.cn; b Chongqing Key Laboratory of Biomedical Analysis (Southwest University) , Chongqing Science & Technology Commission , College of Chemistry and Chemical Engineering , Southwest University , Chongqing 400715 , China; c College of Computer and Information Science , Southwest University , Chongqing 400716 , China; d Beijing National Laboratory for Molecular Sciences (BNLMS) , Key Laboratory of Bioorganic Chemistry and Molecular Engineering of Ministry of Education , Institute of Analytical Chemistry , College of Chemistry and Molecular Engineering , Peking University , Beijing , 100871 , China . Email: lina@pku.edu.cn

## Abstract

The plasmon-induced light concentration (PILC) effect, which has been supposed to be responsible for lots of linear and nonlinear enhanced optical signals such as Raman and high-harmonic generation, is hard to directly observe. Herein, we developed a scattered light based composite-field microscopy imaging (iCFM) system by coupling the oblique and vertical illumination modes, which were adopted in dark- and bright-field microscopy imaging systems, respectively, and through which iCFM system monochromatic background (MCB) images are available, to directly observe the PILC effect in far-field scattering microscopy imaging. Owing to the PILC effect, the scattering signal gain of plasmonic nanoparticles was found to be larger than that of the background, and the imaging visibility of plasmonic nanoparticles was improved by 2.4-fold for silver nanoparticles (AgNPs) and 1.6-fold for gold nanorods (AuNRs). Successful observation of the PILC effect visually together with application in enhanced visibility in cancer cell imaging by this composite illumination system might open an exciting prospect of light scattering microscopy imaging techniques with largely increased visibility.

## Abstract 2

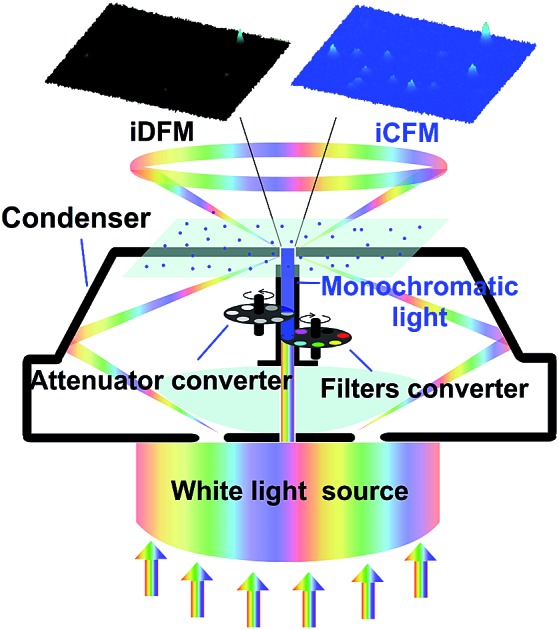
A composite-field microscopy imaging (iCFM) system is constructed to observe the plasmon-induced light concentration (PILC) effect and to enhance imaging visibility.

## Introduction

The plasmon-induced light concentration (PILC) effect,^1^,^2^ an opposite phenomenon to the optical invisible cloak^3^,^4^ which results from the larger extinction cross sections of plasmonic nanocrystals than their geometrical cross sections,^5^ is an effect by which noble metals can unprecedentedly concentrate light into the deep sub-wavelength volumes in close proximity to plasmonic nanoparticles. Enhancement phenomena in nano-optics, including Raman,^6^–^8^ fluorescence,^9^ light absorption in solar energy harvesting,^10^ high-harmonic generation,^11^,^12^ and scattered light of dielectric particles,^2^,^13^,^14^ have been interpreted as involving the PILC effect. Both localized surface plasmon resonance (LSPR) and PILC disclose the strong electromagnetic field attributed to the extensive interaction between the incident light and plasmonic nanoparticles,^15^,^16^ but the former is described mainly in terms of the finite frequency,^17^ while the latter is mainly from the perspective of the broad interaction space.^1^,^2^

Owing to the excellent LSPR scattering property of plasmonic nanoparticles, the dark-field microscopy (DFM)^18^ technique, particularly dark-field microscopy imaging (iDFM), has enabled rapid developments of lots of new applications in the fields of plasmonics.^19^–^27^ Although the high scattering efficiency is closely related to the PILC effect,^2^ there has been difficulty in directly observing the light concentration effect from the scattered light of plasmonic nanoparticles, instead of from some other aforementioned enhanced optical signals, Raman and fluorescence, for instance. To observe the PILC effect and optical space squeezing^1^ in far-field scattering microscopy imaging, both the plane incident light and the corresponding scattered light attributed to this plane incident light must be quantized simultaneously, wherein the bright-field forward scattering imaging mode seems to be appropriate. However, the indistinct scattering signals restricted to the overwhelming brightness of the background in the bright-field mode mean it fails to achieve this goal. Therefore, it remains an experimental challenge to directly observe the PILC effect optically and to make it contribute more to the iDFM technique by further rational design of the existing illumination system.

Herein, we developed a novel composite-field microscopy imaging (iCFM) system by coupling the oblique and vertical illumination modes, which were adopted in dark- and bright-field microscopy imaging systems respectively, to demonstrate the PILC effect experimentally, together with greatly enhanced visibility in plasmon resonance light scattering imaging. It is owing to the PILC effect that the scattering signal gain is larger than that of the background, and the imaging visibility of plasmonic nanoparticles was significantly improved by 2.4-fold for silver nanoparticles (AgNPs) and 1.6-fold for gold nanorods (AuNRs), as demonstrated by structural similarity (SSIM) and RGB analysis. The effectiveness of the iCFM system are further demonstrated by high-pass output of images and extended analysis of monodisperse AgNPs (scattered blue light) and aggregated ones (scattered red light) in cancer cell imaging.

## Results and discussion

### Design of composite-field illumination system

**Fig. 1:**
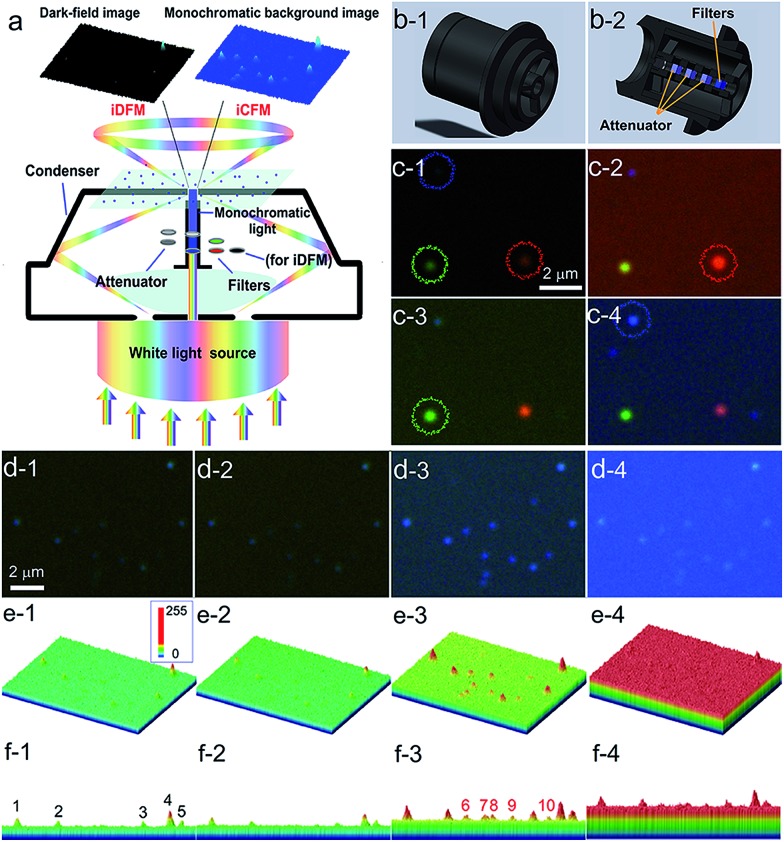
Schematic design of the refitted condenser optical path of the iDFM system and the visibility enhancement. (a) Principle of optical path modification and a schematic diagram of visibility enhancement of iCFM over iDFM. (b) The overall and cross-sectional view of the refitted U-DCD condenser, in which filters and neutral density attenuators were placed along the optical axis. (c) The scattering image of AgNPs of three primary colours under the dark-field (c-1), red background (c-2), green background (c-3) and blue background (c-4), respectively. (d) The scattering imaging of blue AgNPs under dark-field (d-1) and blue background with different intensities adjusted by attenuators (d-2, transmittance 1%, d-3, transmittance 5% and d-4, transmittance 10%). (e) and (f) Three-dimensional displays of the image in d from oblique and side face views.

In order to observe the PILC effect, we designed a composite-field illumination microscopy system by combining the advantages of both dark- and bright-field illuminating features, wherein oblique and vertical illumination modes were adopted, respectively. By using this composite-field illumination microscopy imaging system, monochromatic background (MCB) images could be available. To make the setup, we modified the illumination optical path of a dark-field condenser (U-DCD, Olympus), which had a numerical aperture (NA) of 0.80–0.92, a working distance of 4.52 mm, and a focal length of 11.8 mm (Fig. 1a and b). The bright-field images could be obtained when the central vertical illumination was adopted only (without consideration of the attenuator and the filters), and the dark-field images were obtained when the white light oblique illumination was adopted only (in the top-left of Fig. 1a). If the oblique illumination and the central vertical illumination were adopted together to form the iCFM system, MCB images were obtained (as in the top-right of Fig. 1a). It is the central monochromatic vertical illumination that forms the MCB because it reaches the detection region together with the scattered light (for details, see Fig. S1†). Among these two illumination modes in the iCFM system, the oblique illumination ensured the visibility of the plasmonic nanoparticles, and vertical illumination was used to investigate the PILC effect, wherein the MCB was in fact, the quantifiable plane incident light in the optical space squeezing.

To stimulate the LSPR scattering efficiently, the central vertical beams were converted into monochromatic light with the same energy as the LSPR of plasmonic nanoparticles at first. In a quest for the best illumination effects, a set of neutral density attenuators was mounted to regulate the intensity of the monochromatic illumination light (ESI Section 1 and Fig. S1–S3†). This manipulation opportunely avoided introducing backgrounds into the iCFM with overwhelming brightness as the traditional bright-field imaging did. It was owing to this optical configuration that the light concentration effect of plasmonic nanoparticles could be observed (Fig. 1a).

**Fig. 2:**
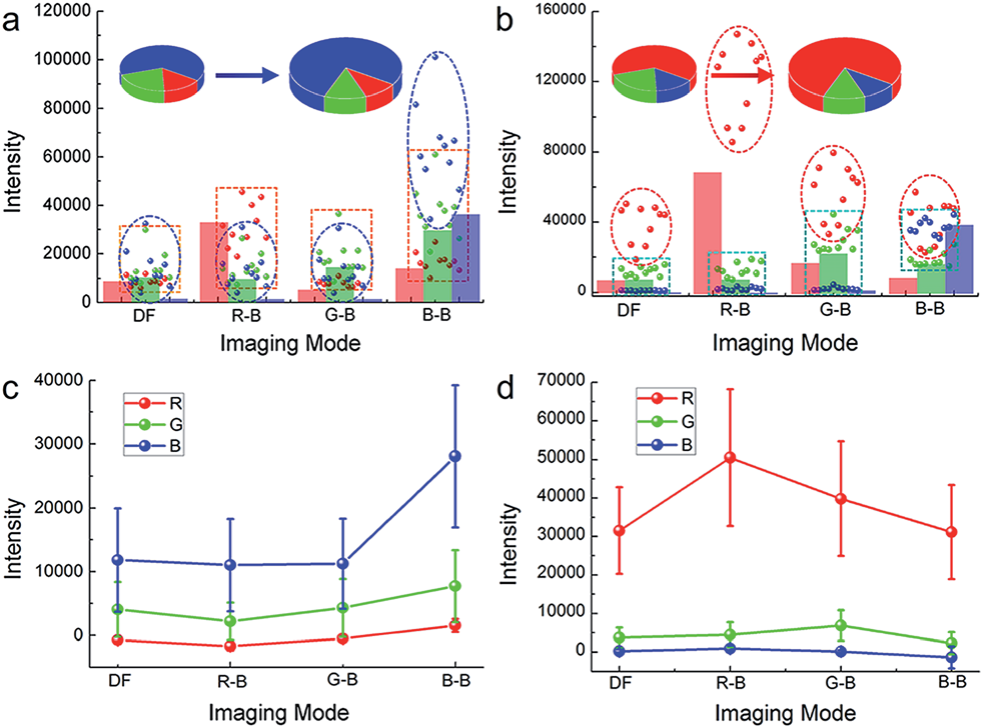
Increased dominant colour value of the nanoparticles and the larger enhancement than background. The colour value of (a) blue AgNPs and (b) red AuNRs in iDFM and iCFM systems. The colour column shows the colour value of the background. The four imaging modes are dark-field (DF), red background (RB), green background (GB) and blue background (BB), respectively. The intensity enhancement of the colour value for (c) blue AgNPs and (d) red AuNRs after deducting the background intensity. Error bars indicate the standard deviation of 10 individual analyzed nanoparticles.

**Fig. 3:**
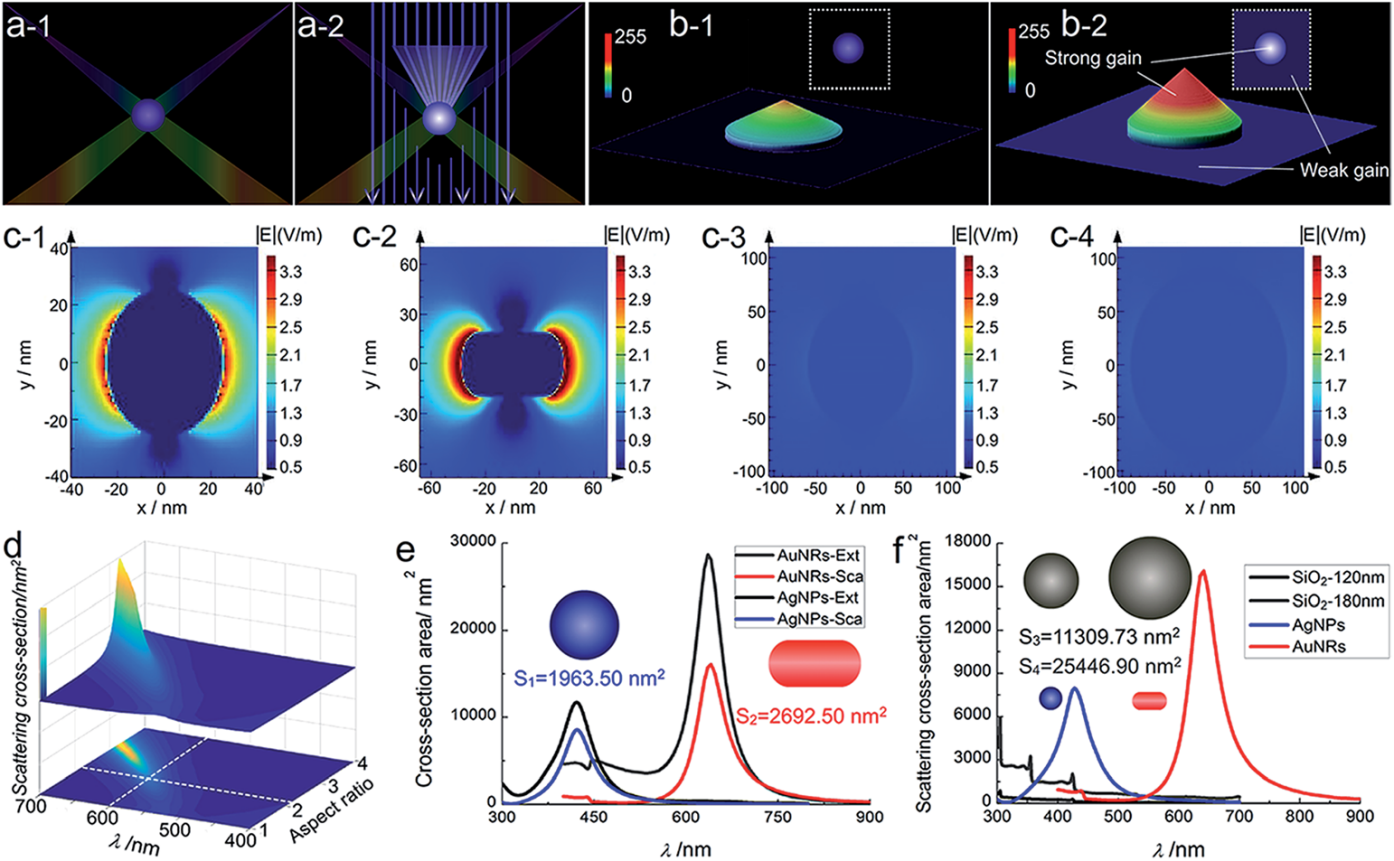
Schematic diagram of light concentration for enhanced visibility and the electric distribution, cross-section area of nanoparticles. Schematic diagram of (a) the light concentration of a plasmonic nanoparticle and (b) the visibility enhancement in the composite illumination optical path (a-2) compared to the dark-field imaging (a-1). (c) Localized electric field distributions of a AgNP (c-1, 50 nm in diameter), AuNR (c-2, 75.4 nm in length, 38.5 nm in diameter), SiO_2_ nanoparticle (c-3, 120 nm in diameter; c-4, 180 nm in diameter) by FDTD simulation. (d) The scattering cross-section of a AuNR determined by the Lorentz model and quasi-static approximation with variable wavelength and aspect ratio, and during the simulation, the cross-section of the nanorod was fixed to be a constant, 2900 nm^2^. (e) Calculated extinction (black line) and scattering (blue line) cross-section of AgNP (50 nm in diameter) and calculated extinction (gray line) and scattering (red line) cross-section of the longitudinal mode of AuNR (75.4 nm in length, 38.5 nm in diameter). (f) Calculated scattering cross-section of SiO_2_ nanoparticles in size of 120 nm (black line) and 180 nm (gray line).

The effectiveness of this refitted condenser could be estimated by using multicolour plasmonic nanoparticles (the preparation and characterization of the used plasmonic and nonplasmonic nanoparticles and the imaging manipulation are described in detail in ESI Section 2 and Fig. S4–S8†). Under the iCFM system, the maximum enhancement was achieved when the illumination energy was matched with the LSPR of the plasmonic nanoparticles (Fig. 1c). With the adjustment of the attenuators, the nanoparticles which were almost invisible in iDFM or in iCFM with inappropriate MCB intensities (no. 6–10 in Fig. 1f-3) were easily observed and detected (Fig. 1d–f).

### Enhanced visibility of MCB images as evaluated by SSIM and RGB analysis

A structural similarity (SSIM) system,^28^ which is independent of image acquisition conditions and observers, is firstly used to evaluate the image quality of both the scattering images of blue AgNPs in iDFM and in iCFM under different blue background intensities or different background colours (the methods and results involved in the SSIM evaluation are shown in detail in ESI Section 3.2†). Because all other SSIM indexes are never higher than 0.60 compared to the images obtained under the optimal conditions (Fig. 1d-3), the monochromatic light in the iCFM system that has a suitable intensity and colour (wavelength range) indeed plays important roles in the visibility enhancement.

Since scattering microscopy imaging is a true-colour imaging technique, both the colour^29^,^30^ and intensity^31^ are important parameters to characterize the image quality. The RGB analysis of the enhanced scattering microscopy imaging visibility under the iCFM system is then performed by using Image-Pro Plus 6.0 Software (ESI Section 3.3†). Owing to the monochromatic light with suitable energy in the iCFM system, the dominant colour value of the plasmonic nanoparticles in scattering images was elevated definitely (Fig. S10†), and it was essentially different from the imaging by naive filtration of the white light source into a monochromatic light source which was accompanied by a reduced scattering intensity (ESI Section 3.4 and Fig. S11–S12†). Besides, the enhanced visibility of the nanoparticles could also not be obtained by simply extending the exposure times (ESI Section 3.5, Fig. S13–S14†).

The blue value proportion of AgNPs had an absolute increase in the blue background imaging, and so did the red value proportion of AuNRs in red background imaging (Fig. 2a and b). If the main colour value in iDFM was regarded as 1, the blue colour value of AgNPs and the red colour value of AuNRs in iCFM had an increase of 137.5% and 60.1%, respectively (Fig. 2c and d), revealing that the iCFM system indeed achieved recognizable imaging visibility promotion.

### Identification of the PILC effect responsible for the visibility enhancement

In order to identify the PILC effect of noble metal nanoparticles responsible for the enhanced imaging visibility, dark-field and MCB scattering images of nonplasmonic SiO_2_ nanoparticles as controls were also obtained. The scattering light colour of SiO_2_ nanoparticles is not monochromatic but polychromatic, which corresponds with their broad scattering spectra which cover almost the entire visible wavelength region (ESI Section 2.3, Fig. S8†). Different from the plasmonic nanoparticles, SiO_2_ nanoparticles don't show any enhancement of the imaging visibility and integral scattering intensity^31^ under the iCFM system (ESI Section 4.1, Fig. S15–S16†).

**Fig. 4:**
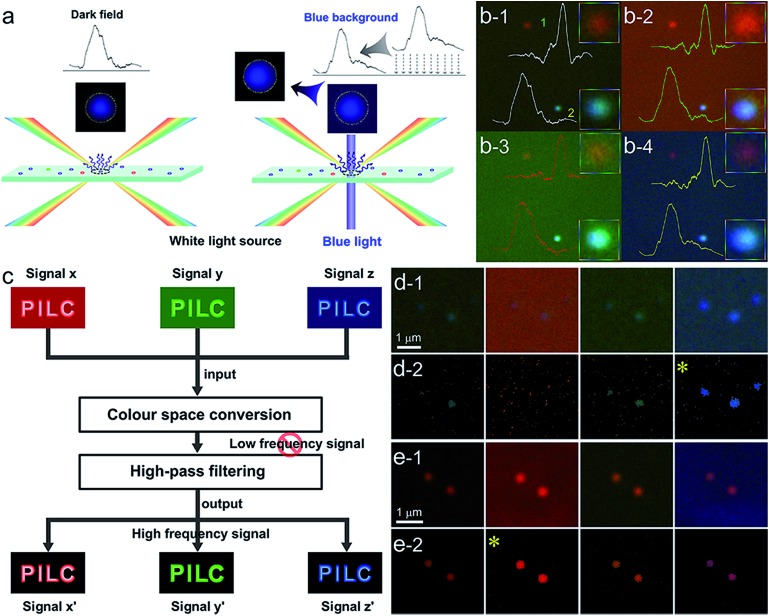
High fidelity of the scattering spectra and high-pass output of scattering images. (a) Schematics of the increased scattering intensity and substantially constant wavelength of the blue nanoparticles under blue background imaging in iCFM compared to iDFM. (b) Experimental data of the enhanced intensities of blue AgNP and red AuNR in iDFM and iCFM, and the corresponding spectra. (c) Diagram of high-pass output of the scattering images. (d) and (e) High-pass output of the blue AgNPs and the red AuNRs as shown in Fig. 2a and c.

Obviously, the imaging visibility enhancement under the iCFM system is inextricably linked to two factors, the PILC effect of noble metal nanoparticles and the monochromatic light with the same energy as the LSPR. The plasmon-induced concentration of this monochromatic light into the adjacent regions of the plasmonic nanoparticles greatly enhanced the scattering intensity rather than simply superimposing the monochromatic light intensity onto the scattering image of the plasmonic nanoparticles (Fig. 3a and b). The PILC effect could also be identified by the enhanced electric field distributions around the plasmonic nanoparticles as calculated by the finite-difference time-domain (FDTD) method (for detail, see ESI Section 4.2†). As a comparison, no obvious enhancement could be found around the SiO_2_ nanoparticles (Fig. 3c-1–c-4).

For nanoparticles, the scattering efficiency, which is a function of the incident wavelength *λ*, could be calculated as the ratio of the extinction cross-section to the geometrical cross-section (namely the physical cross-section, *S*).^32^ The scattering efficiencies of AgNPs^33^ (eqn (S1) in ESI Section 4.3†) and AuNRs^34^ (eqn (S2)–(S4) in ESI Section 4.3†) were both wavelength-dependent, and that of AuNRs was also closely related to the aspect ratio (Fig. 3d). When the aspect ratio of the nanorod was about 2, the maximum scattering wavelength was at ∼600 nm (red colour), which was similar to and slightly shorter than that of the used AuNRs (∼630 nm).

The used AgNPs and AuNRs had large scattering efficiency (4.35 and 5.97 at the maximum scattering wavelength for AgNPs and AuNRs), and at nearby LSPR wavelengths (about 50 nm width range), they also had a high scattering efficiency much larger than 1 (Fig. 3e and f). Therefore, they could have enhanced visibility larger than the monochromatic light induced background, while the nonplasmonic SiO_2_ nanoparticles, which had a much smaller scattering cross-section area and lower scattering efficiency (at least 2 orders of magnitude lower than the plasmonic nanoparticles), did not show obvious visibility enhancement. The scattering signal of plasmonic nanoparticles might have a maximum enhancement (equal to the maximum scattering efficiency) in an ideal situation, for instance, when only plane incident light illumination existed (a situation that the proportion of the scattering contributed by the plane incident light illumination was the largest theoretically). Under the iCFM, the ratio of the blue value gain of the AgNPs to that of the corresponding MCB was 1.54, and it was 1.18 for AuNRs. This decreased gain compared to the theoretical values could be attributed to the existing PILC effect of the plasmonic nanoparticles in the dark-field oblique illumination, which was difficult to measure experimentally but had played important roles in the scattering intensity and the imaging visibility.

Herein, the realized higher scattering intensity enhancement efficiency of AgNPs (2.38) than that of AuNRs (1.60) might be attributed to the smaller original blue value intensity of AgNPs than the original red value intensity of AuNRs (Fig. 3c and d) in iDFM. In addition, the spectral response function of DP72 CCD showed that it had a lower collection coefficient (∼73.5%) at the ∼475 nm (maximum response wavelength of blue colour) than at ∼620 nm (maximum response wavelength of red colour),^35^ therefore, a similar enhancement in scattering intensity of the plasmonic nanoparticles might lead to a higher visibility increase in blue background imaging than in red background imaging.

### High fidelity of the scattering spectra and high-pass output of image

As known, LSPR scattering is elastic Rayleigh scattering, and the energy of the monochromatic light under optimal imaging conditions of iCFM mainly distributes around the level of LSPR wavelength, so only the scattering intensity at the LSPR wavelength could be enhanced effectively. During this process, the LSPR peak should retain its inherent waveband (Fig. 4a), which was similar to the LSPR excited by the white light source in iDFM. The experimental results were in good agreement with this deduction, and the LSPR spectra of both the blue and red nanoparticle indeed retained a similar waveband (Fig. 4b). Both the blue value and the total RGB value of the AgNP were largest in the blue background image and they had an addition of 147.0% and 80.13% as compared to that in dark-field microscopy image. The AuNR showed the same behavior and the corresponding addition of the red value and total RGB value were 100.4% and 78.37%, respectively (ESI Section 5, Fig. S17–S18†). To facilitate the spectra scanning and collection, an AgNP with high scattering intensity and blue value intensity was selected for the spectra determination, and as a result, the enhancement efficiency of this AgNP was changed to a similar level as the AuNR.

Nowadays, high-pass output of the scattering image^36^,^37^ has become an effective strategy to obtain a new related image in which the useful information is retained and the useless information is deducted. A notable advantage of the high-pass output strategy is the high information fidelity, which is always distorted in the background subtraction process by simple contract adjustment, although a high signal to noise ratio might be obtained (Fig. S19†). In such case, we applied this strategy to extract the imaging information of plasmonic nanoparticles from the background (the word “PILC” which is high frequency signal is retained and the MCB which is low frequency signal is reduced) by two simple steps, colour space conversion and high-pass filtering (Fig. 4c). The high-pass output results of the scattering images of blue AgNPs and red AuNRs under different imaging modes (Fig. 4d and e) showed that the imaging visibility was indeed the best in iCFM when the monochromatic light had the matched energy, and this result was consistent with the SSIM and RGB analysis results. Besides the visibility enhancement, the excellent colour fidelity capability of the iCFM technology had also been revealed.

### Scattering imaging of cancer cell-plasmonic nanoparticles with iCFM

**Fig. 5:**
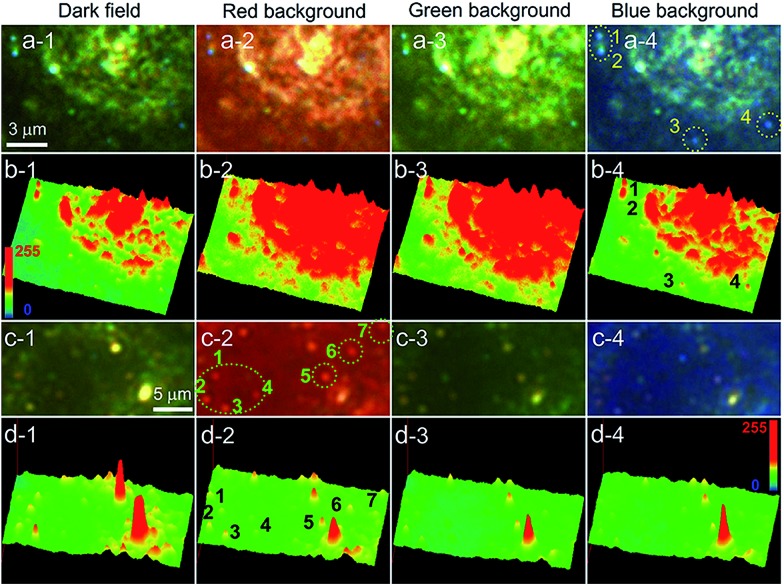
Imaging of AgNPs protected by BSA and AgNPs without protection after they are incubated with Hep-2 cancer cell by iDFM and iCFM system. (a) Scattering images of BSA protected blue AgNPs after they are incubated with Hep-2 cancer cell in dark-field and MCB imaging. (b) The corresponding three-dimensional mode of the scattering images showed in (a). (c) Scattering images of aggregated red AgNPs without protection of BSA after they are incubated with Hep-2 cancer cell in dark-field and MCB imaging. (d) The corresponding three-dimensional mode of the scattering images showed in (c).

Finally, we investigated the use of the iCFM system to image the AgNPs in cancer cell imaging which always has high scattering backgrounds. The AgNPs protected by the bovine serum albumin (BSA) and the unprotected AgNPs showed significant difference in the dispersion state and LSPR scattering properties. The used AgNPs were mostly spheres and scattered blue light even after modification with BSA (ESI Section 6 and Fig. S20†). Even after incubation with human laryngeal epithelial carcinoma (Hep-2) cells, the BSA protected AgNPs still scattered blue light, suggesting these AgNPs maintained a decentralized state (Fig. 5a and b). Both the scattering images (Fig. 5a) and the three-dimensional display mode (Fig. 5b) showed the visibility of the dispersed AgNPs was optimal in the blue background imaging under iCFM, especially for the AgNP (no. 3, Fig. 5a-4) which had a weak scattering intensity and poor imaging visibility in the iDFM.

After similar operations, except that the used AgNPs were the unprotected ones, the AgNPs scattered red light. By comparison of the red dots and the retained blue dots, it could be deduced that the red dots could be attributed to the aggregation state of the AgNPs (Fig. S21j†). In addition, the aggregation of the AgNPs could be clearly confirmed from the visible precipitation when the concentration of the AgNPs contacting with the 1640 cell culture media was larger, however, the protected AgNPs remained dispersed. The imaging of the unprotected AgNPs in the Hep-2 cancer cell showed the optimal visibility of the red aggregates was observed in the red background imaging (Fig. 5c). Imaging visibility analysis at the single nanoparticle level revealed that the blue value of dispersed AgNPs and the red value of the aggregated AgNPs was obviously beyond the background value under the corresponding same coloured background imaging (Fig. S22a4 and b2†). These results demonstrated this iCFM technology could be applied in the imaging analysis of complex biological samples and systems.

## Conclusions

We have shown an iCFM system for the direct observation of the PILC effect and achieving visibility enhancement of the plasmonic nanoparticles in far-field scattering imaging. It's worth stressing that the high enhancement efficiency of the scattering intensity and visibility was attributed to the large scattering efficiency and the PILC effect of the plasmonic nanoparticles, and thus it is suitable for the scattering imaging of plasmonic nanoparticles, which are the most commonly used probes in scattering imaging.

Because of higher scattering response sensitivity of plasmonic nanoparticles than organisms inside living cells at the visible light region,^38^ the iCFM system can be applied in the analysis of complex biological samples and systems. The PILC effect and iCFM system introduces the best visibility enhancement when the energy of the monochromatic vertical beams is matched to the LSPR of the nanoparticles, so iCFM can amplify the degree of the signal change if the LSPR of nanoparticles is regulated in reaction monitoring and analysis. In addition, some new emerging plasmonic nanomaterials, such as heavily-doped colloidal semiconductor,^39^ graphene^40^ and J-aggregate dyes,^41^ are no longer limited to the traditional noble metals and the thin films of J-aggregate dyes has been confirmed to have an excellent PILC effect,^41^ suggesting this iCFM system might have wide applications.

Future attempts may involve the development of a refit light path on the basis of an oil-immersed condenser with a higher NA value and the equipment of the iCFM system with a monochromatic illumination light which has a continuously variable intensity, in pursuit of super-resolution techniques in far-field optical scattering imaging.

## Experimental

### Scattering imaging instrument

All the scattering imaging was performed with a BX51 optical microscope (Olympus, Japan) equipped with a DP72 single chip true-colour charge coupled device (CCD) camera. The light source used in the iDFM and iCFM was the same one, that was a 100 W halogen light source (U-LH100-3). In the imaging process, the power of the light source was kept at the maximum. The scattering spectra of the blue and red single nanoparticles were measured by a connected spectrograph (MicroSpec-2300i, Roper Scientific) and intensified CCD camera (PI-MAX, Princeton Instrument) to the BX51 dark-field optical microscope. The gain value was kept as 100 to get a good signal-to-noise ratio and strong signal intensity. Dark-field condensers (U-DCW, oil-immersed, NA = 1.2–1.4; U-DCD, dry type, NA = 0.80–0.92) and a 100× oil-immersed and a 40× non-oil-immersed object lens were used in the imaging. The refit of the optical path was on the basis of the U-DCD condenser and the process in detail and the used optics are shown in the ESI Section 1.†

### Characterization and date analysis

The extinction spectra of the nanoparticles were measured with a Hitachi U-3010 spectrophotometer. Scanning electron microscopy (SEM) images were measured with a Hitachi S-4800 field emission scanning electron microscope. The scattering intensity and the RGB analysis were accomplished by the Image-Pro Plus 6.0 Software and the analysis methods are provided in the ESI Section 3.3.†

## Supplementary Material

Supplementary information
