# Supplementary material for: Plasmon-induced light concentration enhanced imaging visibility as observed by a composite-field microscopy imaging system
Source: Chem Sci. 2016 Apr 25;7(8):5477–83. doi: 10.1039/c6sc01055e (PMC6021787; doi:10.1039/c6sc01055e)
Supplement: Supplementary file 1 [file SC-007-C6SC01055E-s001.pdf]

Electronic Supplementary Material (ESI) for Chemical Science.
This journal is © The Royal Society of Chemistry 2016

Electronic Supplementary Information (ESI) for:

Plasmon-induced light concentration enhanced imaging visibility as observed by a composite-field microscopy imaging system

Peng Fei Gao,[a] Ming Xuan Gao,[b] Hong Yan Zou,[a] Rong Sheng Li,[a] Jun Zhou,[c] Jun Ma,[b] Qiang Wang,[a] Feng Liu,[d] Na Li,*[d] Yuan Fang Li[b] and Cheng Zhi Huang*[a,b]

This file includes:

Supplementary Information Sections 1 to 6.

1. Optical path refit of the condenser for iCFM system (**Figure S1, Table S1-S2, Figure S2-S3**).

2. Synthesis, characterization and iDFM of the used nanoparticles.

2.1. Synthesis and characterization of the used plasmonic nanoparticles (**Figure S4**).

2.2. Synthesis and characterization of the SiO_2 nanoparticles (**Figure S5**).

2.3. Scattering properties of the AgNPs, AuNRs and the SiO_2 nanoparticles (**Figure S6-S8**).

3. Imaging of plasmonic nanoparticles by the newly developed iCMF system.

3.1. Imaging operation by iCMF system (**Figure S9**).

3.2. Enhanced visibility of MCB images as evaluated by SSIM.

3.3. Enhanced visibility evaluated by RGB analysis (**Figure S10**).

3.4. Scattering imaging of AgNPs with a filtered monochromatic light source (**Figure S11-S12**).

3.5. Three-dimensional model and high-pass output of the scattering images (**Figure S13-S14**).

4. PILC responsible for the visibility enhancement.

4.1. Scattering imaging of SiO_2 nanoparticles with the iCMF system (**Figure S15-S16**).

4.2. Finite-difference time-domain (FDTD) simulation.

4.3. Wavelength-dependent scattering efficiency of AgNPs and AuNRs (**Equation S1-S4**).

5. Single nanoparticle spectra of AgNPs under different imaging modes (**Figure S17-S19**).

6. Hep-2 Cells-AgNPs scattering imaging with iCFM system (**Figure S20-S22**).

Supplementary references 1-9.

SUPPLEMENTARY INFORMATION SECTIONS 1 TO 6.

1. Optical path refit of the condenser for iCFM system.

Prior to the refit of the illumination optical path of dark-field microscopy imaging (iDFM) system to build the composite-field microscopy imaging (iCFM) system, we firstly identified the thickness of the embowed refractive element in the axis of U-DCD dark-field condenser. After ensured the thickness was ~1.0 mm (noted by red double slash in Fig. S1a) which was ease of casting and might bring little influence to the concentrating effect of the condenser, we dug a round hole ($\Phi = 5$ mm) at the arc of the embowed refractive element (Fig. S1c). In order to adjust the introduced light to a monochromatic light with a controllable intensity and colour, a filter and a set of neutral density attenuators were installed into the inner of the U-DCD condenser through the fixation of the custom optical bracket (Fig. S1d and e).

The optical lens ($\Phi = 6.8$ mm) were placed in a pipe which had internal threaded and was connected to outer cylinder by three symmetrical distributed connecting arm. Between the adjacent connecting arms there was 120 degree intervals to reduce the light intensity difference on the light ring of the oblique dark-field illumination as much as possible. After the excavation of the hole, the dark-field effect was obtained by a black lens in the same size and was also fixed to a circular metal frame ($\Phi = 7.0$ mm) in the same manner with other optical lens (Fig. S1e). The optical parameters of the optical lens were showed in Table S1 and S2.

With the refit condenser, monochromatic light was indeed obtained as shown in Fig. S2. To better display the illumination principle of the refit optical path, we then make the formed monochromatic background visible with the help of a common coverslip and the light path schematic diagram was showed as Fig. 3a. It was the “Tyndall effect” of the impurities on the coverslip mapped out the formed composite spot, containing a white focal point and a round monochromatic spot (Fig. S3c-e). In the central, the inherent white focal point of dark-field illumination was also seen, suggesting the introduced monochromatic illumination brought a superimposed imaging effect of a monochromatic field (Fig. S2).

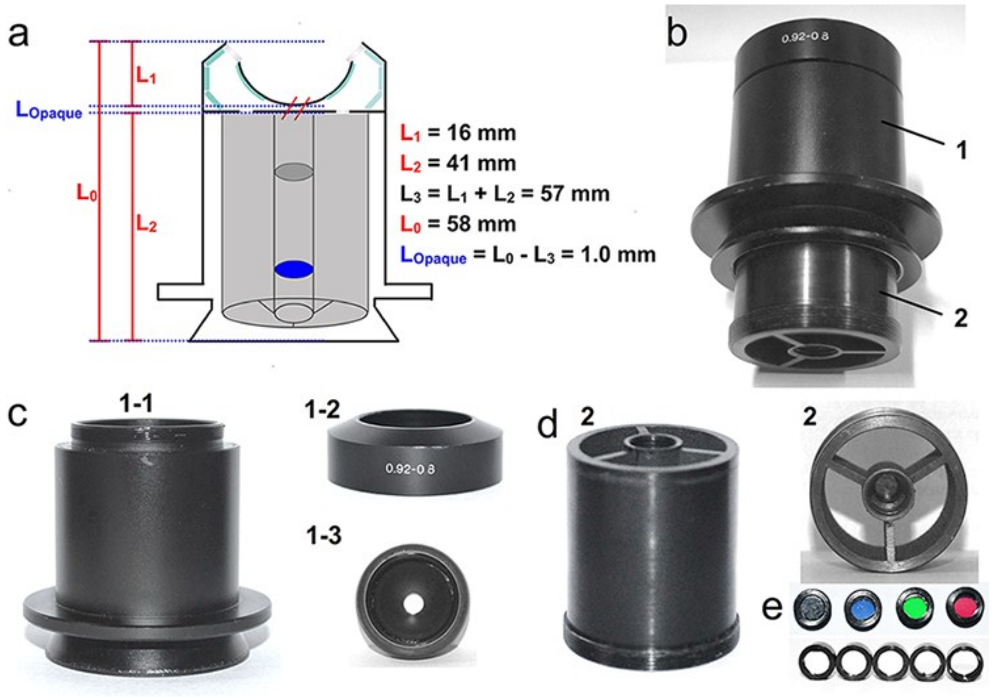

Figure S1 Constituent elements of the iCFM condenser. (a) Schematic cross-section and size parameters of the U-DCD dark-field condenser. It's in the position of the red double slash along the optical axis that a round hole (Φ = 5.0 mm) was punched as the showing of 1-3 in (c). (b) Photos of the U-DCD condenser (1, including 1-1, 1-2 and 1-3 as shown in (c)) and the custom optical bracket installed inside the condenser (2, as shown in (d)). (c) Three split components of the condenser, (1-1) the lens barrel, (1-2) the lens cap and (1-3) the refractive element. The monochromatic light was introduced through the round hole on (1-3). (d) Photos of the optical bracket (2). (e) Photos of the attenuators and filters, which are placed on the central of (2) in order to obtain monochromatic illumination light.

Table S1 | Optical parameters of the bandpass filters used to obtain the monochromatic background light.

Filter colour	Central wavelength	Bandwidth	Transmission at central wavelength
blue	470 nm	25.0 nm	> 85%
green	535 nm	77.8 nm	> 90%
red	650 nm	95.5 nm	> 90%

Table S2 | Actual transmission of the neutral density attenuator in the wavelength range of 400-800 nm.

Attenuator	Actual transmission in 400-800 nm
1%	0.0% < T < 1.7%
5%	4.5% < T < 7.7%
10%	9.8% < T < 10.3%

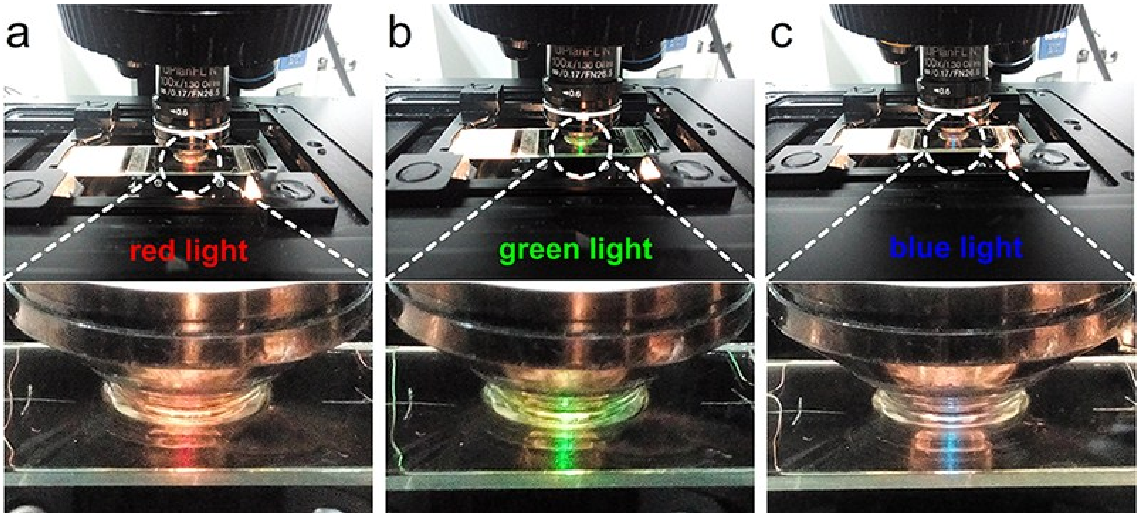

Figure S2 Introduced monochromatic light in the optical axis under iCMF system. (a) Photos of the introduced red light along the optical axis. Coloured optical beams can been seen clearly from the enlarged pictures. (b), (c) Photos of the introduced green and blue light along the optical axis.

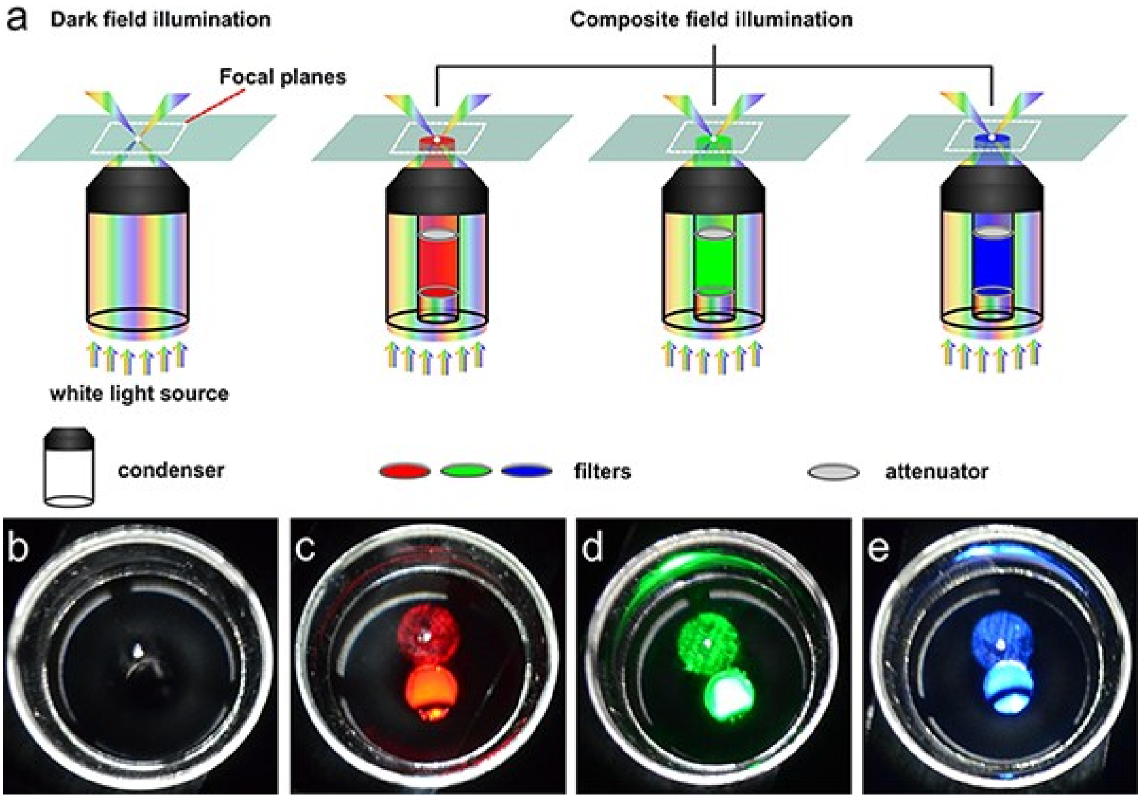

Figure S3 Composite spot formed on the focal plane in iCMF system. (a) Schematic diagram of the different focal planes of dark-field illumination (a white focal point) and the composite-field illumination (a composite spot, contains a white focal point and a round monochromatic spot). The condenser is illuminated by a white light source from the bottom, and the focal plane is visible on the coverslip

attributed to the "Tyndall effect". (b) Actual observation of the white focal point under dark-field illumination. (c)-(e) Actual observation of the composite spots under the composite-field illumination.

2. Synthesis, characterization and iDFM of the used nanoparticles.

2.1. Synthesis and characterization of the used plasmonic nanoparticles.

The silver nanoparticles (AgNPs) coated by sodium citrate were prepared according to the previous report[1] with some modifications. Briefly, glycerol/water mixture (20 vol% glycerol, 50 mL) was stirred violently in a 150 mL conical flask. After the solution was heated up to 95°C, 8.5 mg silver nitrate (Shenbo, Shanghai, 99.8%) was added to the solvent and 0.8 mL 3% sodium citrate (wt%, Ruijinte, Tianjin, 99.0%) was added after a 1 min interval. Stop heating after the solvent was stirred for another 15 min and keep stirring until the solvent cooled down to the room temperature. These operations were for the preparation of AgNPs in the size of ~30 nm. AgNPs of large size of ~41.8 nm and 52.0 nm could be prepared by adding of $AgNO_3$ and sodium citrate for a regrowth procedure (Fig. S4a and b).

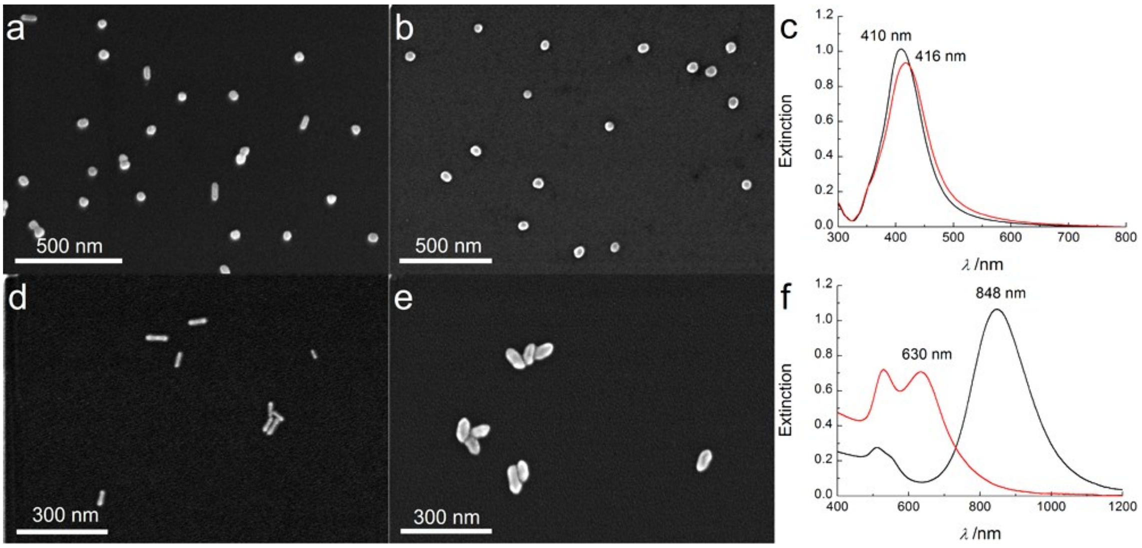

Figure S4 Morphology and spectral characterization of the used plasmonic nanoparticles. (a) SEM images of the sodium citrate coated AgNPs in size of 41.8 nm, most of them are spherical and few are rod-like. (b) SEM images of the sodium citrate coated AgNPs in size of 52.0 nm and most of them are spherical. (c) Extinction spectra of the AgNPs, and the black and red lines correspond with the order in the above SEM images in (a) and (b). (d) SEM images of the prepared AuNRs with large aspect ratio (52.0 nm in length, 15.8 nm in diameter) which is used as the seeds of the overgrowth. (e) SEM images of the AuNRs after the overgrowth (75.4 nm in length, 38.5 nm in diameter). (f) Extinction spectra of the

gold nanoparticles and the black, red and blue lines also correspond with the order in the SEM images in (d) and (e).

The used gold nanorods (AuNRs) in Fig. 2c were prepared with a regrowth method[2]. The seeds used in the regrowth process was firstly prepared according to the previous reports[3] with little adjustments. For a typical synthesis, 0.5 mL of 10 mM $HAuCl_4$ (Sinopharm, Shanghai, Gold content 47.8%) solution was mixed with 15 mL of 0.1 M CTAB (Sinopharm, Shanghai, 99.0%) solution. Under vigorous stirring, 1.2 mL of ice-cold 10 mM $NaBH_4$ (Guangfu, Tianjin, 98.0%) solution was added to form the brown seeds. In a period of 2-5 hour, the seeds was used to prepare the AuNRs. To a mixture solution containing 100 mL of 0.1 M CTAB and 5 mL of 10 mM $HAuCl_4$, 1 mL of 10 mM $AgNO_3$ and 2 mL of 0.5 M H_2SO_4 were added. Then, 0.8 mL of 0.1 M ascorbic acid (Fluka, USA, 99.0%) as the reducing agent was added. After the solution faded, 0.36 mL of the prepared seeds above was added. The solution was kept intact for 24 hours after firstly stirred vigorously for 30 seconds. The prepared AuNRs had an aspect ratio mainly larger than 4 and the longitudinal absorption wavelength was at 848 nm in Fig. S3d. For the AuNRs with such large aspect ratio and long wavelength could not be monitored with the Olympus BX-51 dark-field microscope, so AuNRs with short wavelength in the visible region and strong scattering intensity were prepared by regrowth method.

Before the regrowth of the AuNRs, a stock solution was needed to be prepared by mixing 76 mL of 0.1 M CTAB solution, 3.2 mL of 10 mM $HAuCl_4$ solution, 0.48 mL of 10 mM $AgNO_3$ solution and 0.51 mL of 0.1 M ascorbic acid. 5 mL of the seeds, namely the above prepared AuNRs with large aspect ratio, was mixed with 0.075 mL 10 mM cysteine which was used to induce the overgrowth preferentially on the side surfaces of nanorods because of their selectively absorption to the ends of the AuNRs. The used AuNRs in in Fig. 2c were prepared by mixing of the above seeds and 40 mL of the stock solution. The extinction spectra showed in Fig. S4f revealed the blue shift of the longitudinal absorption wavelength to the visible region.

2.2. Synthesis and characterization of the SiO_2 nanoparticles.

The SiO_2 nanoparticles in size of 118.1 nm were prepared according to the previous reported modified Stöber method and with some modification[4]. Briefly, to 50 mL ethanol solution (70%, v/v) containing 500 mg polyvinylpyrrolidone (PVP, M_w = 55 000, Sigma, America), 3 mL ammonium hydroxide solution (26-28%) and 1.5 mL Tetraethyl orthosilicate (TEOS) (Sigma, America, 99.0%) was added under vigorous stirring, and stirred for 1 h to form a homogeneous colloidal solution.

The SiO_2 nanoparticles in size of 180.5 nm were prepared through a regrowth progress[5]. Firstly, 20 mL of the prepared SiO_2 nanoparticles in size of 118.1 nm was centrifuged and redispersed into 4 mL deionized water. To the above SiO_2 seeds, 20.8 mL of 2-propanol, 300 mg PVP and 0.5 mL TEOS were added. Under stirring, 0.75 mL ammonium hydroxide solution was added, and the after 2 h the regrowth progress was finished.

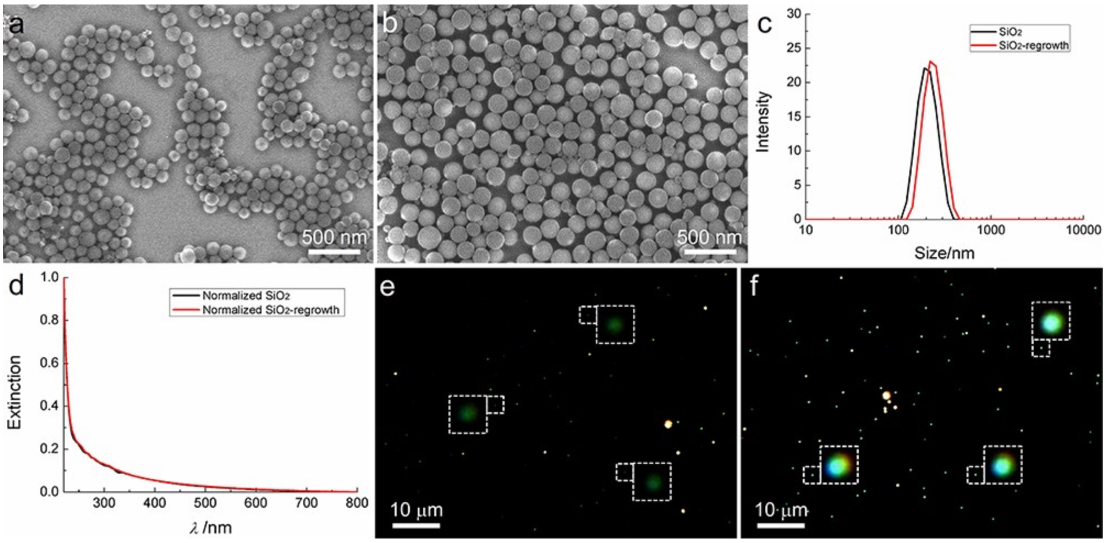

Figure S5 Morphology and spectral characterization of the SiO_2 nanoparticles. (a) SEM images of SiO_2 in size of 118.1 nm. (b) SEM images of the SiO_2 after regrowth in size of 180.5 nm. (c) Dynamic light scattering measurement results of the SiO_2 nanoparticles (black line) and the SiO_2 nanoparticles after regrowth process (red line with an increased size). (d) Normalized extinction spectra of these two SiO_2 nanoparticles. (e) Representative scattering image of the SiO_2 nanoparticles in size of 118.3 nm and the enlarged images of three particles. (f) Representative scattering image of the SiO_2 nanoparticles in size of 180.5 nm and the enlarged images of three particles.

The size and morphology of these two SiO_2 nanoparticles were characterized by SEM and dynamic light scattering (DLS) measurement (Fig. S5a-c). The average size of these two SiO_2 nanoparticles were 118.1 ± 14.6 nm and 180.5 ± 14.0 nm. From the extinction spectra, both of these SiO_2 nanoparticles had no characteristic peaks in the whole visible area (Fig. S5d). The scattering imaging intensity of the SiO_2 nanoparticles in size of 118.1 nm was weak, and that of the SiO_2 nanoparticles in size of 180.5 nm was much stronger and suitable as the control nonplasmonic nanoparticles (Fig. S5e and f).

2.3. Scattering properties of the AgNPs, AuNRs and the SiO_2 nanoparticles.

The scattering spectra of the single plasmonic or nonplasmonic nanoparticles were measured by connecting a spectrograph (MicroSpec-2300i, Roper Scientific) and an intensified CCD camera (PI-MAX, Princeton Instrument) mounted onto the BX51 dark-field microscope. Both 10 nanoparticles of the AgNPs and AuNRs were measured and their corresponding image and spectra was showed in Fig. S6 and S7. The average maximum scattering wavelength of AgNPs was about 453.6 nm and that of the AuNRs was about 631.8 nm. Both of these maximum scattering wavelength were consistent with the blue and red colour. The measured spectra of these plasmonic nanoparticles suggested that the blue and red filters was suitable.

Different from the scattering spectra of AgNPs and AuNRs with sharp peaks, the scattering spectra of SiO_2 nanoparticles in size of 180.5 nm was bread peak and was consistent with their scattering images with mixed colours in Fig. S5f (Fig. S8).

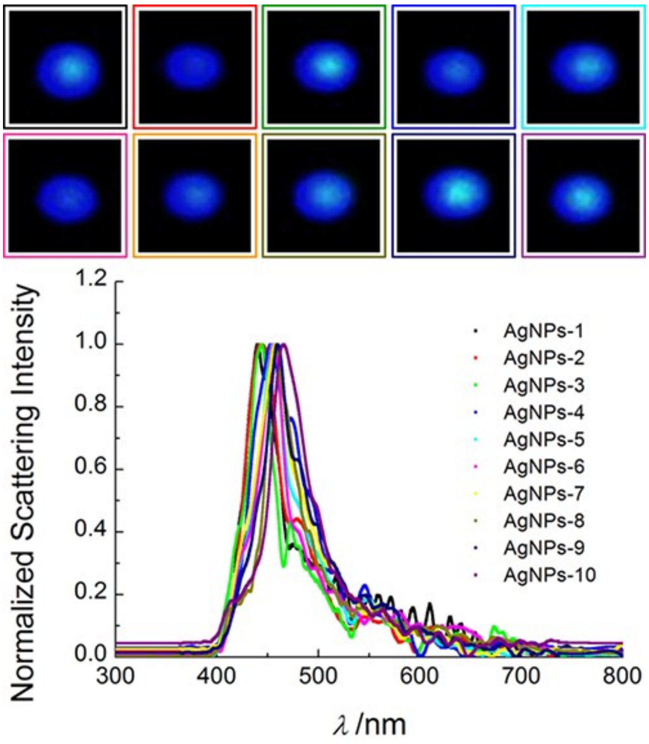

Figure S6 Single nanoparticle scattering spectra of the used AgNPs. Normalized scattering spectra of 10 single AgNPs (52 nm) and their corresponding scattering images.

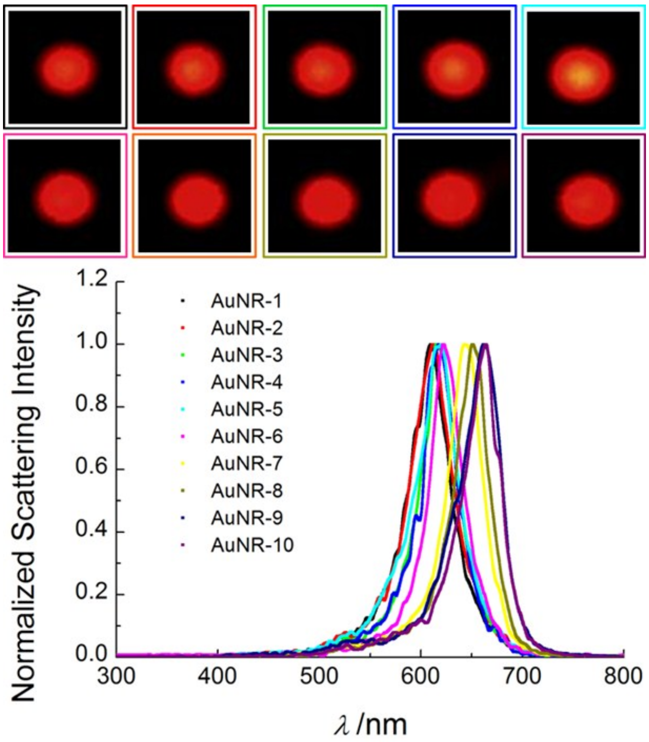

Figure S7 Single nanoparticle scattering spectra of the used AuNRs. Normalized scattering spectra of 10 single AuNRs (75.4 nm in length, 38.5 nm in diameter) and their corresponding scattering images.

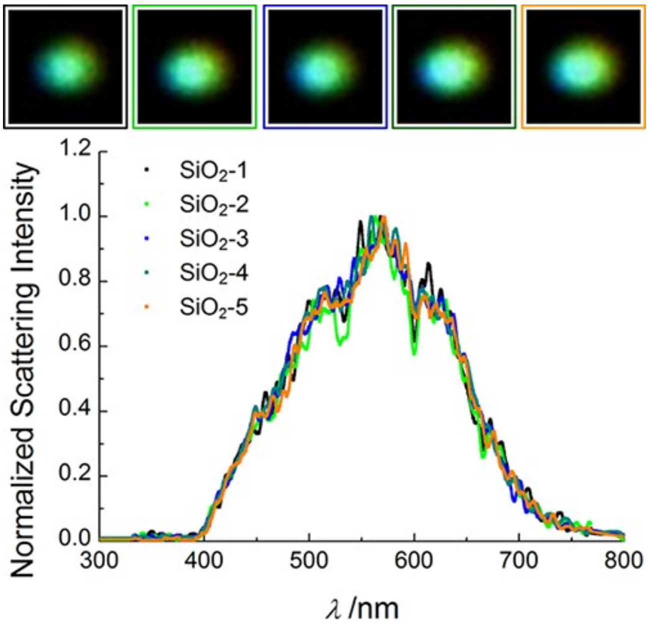

Figure S8 Typical single nanoparticle scattering spectra of the SiO_2 nanoparticles. Normalized scattering spectra of 5 single SiO_2 nanoparticles and their corresponding scattering images.

3. Imaging of plasmonic nanoparticles by the newly developed iCMF system.

3.1. Imaging operation by iCMF system.

The iCFM system based on the refitted condenser was used in the same manner with the original U-DCD condenser and the colour and intensity of the introduced monochromatic light were controlled by changing of the optical lens showed in Section 1, as a result, the nanoparticles, for example of the blue AgNPs, could be imaged with different imaging modes as Fig. S9 showed. In the imaging of the same nanoparticles under different imaging modes, the objective lens and the used homemade sample trough made by glass slide and coverslip[6] were kept standing motionless to maintain the focal length along the whole imaging process. No matter imaging with the oil-immersed or the dry type objective lens, the objects above the glass slide remained intact.

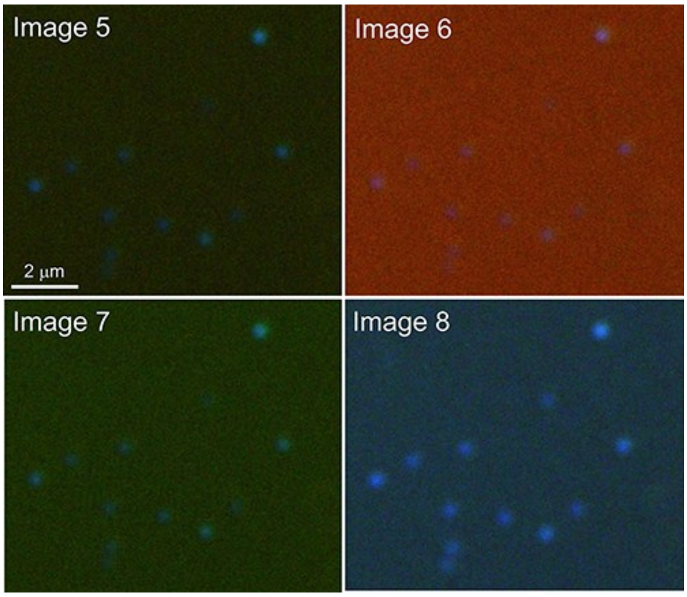

Figure S9 Scattering imaging of AgNPs under different imaging modes. Dark-field image of AgNPs (Image 5). Scattering images of AgNPs with the red, green and blue coloured background imaging mode (Image 6-8).

3.2. Enhanced visibility evaluated by RGB analysis.

Structural similarity (SSIM) system[7] can impersonally reflect the image quality, mainly from the perspective of the luminance measurement (μ_x, μ_y), the contrast measurement (δ_x, δ_y), the luminance comparison (l(x, y)), the contrast comparison (c(x, y)), and the structure comparison (s(x, y)) of two nonnegative image signals, wherein the x and y indicate two different images used for comparison. If we choose an image with perfect luminance and contrast as the reference based on human net eye visualization, the SSIM index can serve as a quantitative measurement of the quality of any other image needing for evaluation. An SSIM index lower than 0.80 reveals an easily distinguished image quality difference, and a value lower than 0.70 reveals an significant difference[7].

The SSIM index reflecting the quality of the scattering images of blue AgNPs under different blue background intensities (Fig. 1d 1-4 represented as Image 1-4) thus could be evaluated by setting Image 3 as the reference. The SSIM index operation results of SSIM (Image 1, Image 3), SSIM (Image 2, Image 3) and SSIM (Image 4, Image 3) could be available with the values of 0.5393, 0.5499 and 0.6030 (according to equation (13) in

reference 24), respectively, revealing that none of Image 1, 2 and 4 had a good quality and visibility.

The SSIM index was then used to evaluate the image quality of the scattering images of blue AgNPs under dark-field and composite-field and it showed that images under the monochromatic light of same colour had best image quality and visibility (Fig. S9). If Image 8 was set as the reference according to the subjective evaluation, the SSIM index of SSIM (Image 5, Image 8), SSIM (Image 6, Image 8) and SSIM (Image 7, Image 8) was 0.5556, 0.3961 and 0.5705, respectively, revealing that none of Image 5, 6 and 7 had a good quality and visibility.

3.3. Enhanced visibility evaluated by RGB analysis.

The enhanced visibility of the used blue AgNPs (52.0 nm) and red AuNRs (75.4 nm in length, 38.5 nm in diameter) can be recognized from the increased blue or red value. The RGB analysis of the scattering images is accomplished by the "Line Profile" function of Image-Pro Plus 6.0 Software. For an image, perform the "Line Profile" function options, and then adjust the position of the line to pass through the nanoparticles to be analyzed. The RGB distribution along the line can be obtained in the form of dimensional curve, and the date of the curve can be exported out. For the images of the same nanoparticles in different imaging modes, the line distribution of the same position can be obtained. This statistical function is convenient for the comparison of the imaging effect under different imaging modes.

The RGB line distribution analysis of two nanoparticles in a same line were achieved in the same manner with the single nanoparticle and just extended the line length to pass through the two nanoparticles (Fig. S10). Although the monochromatic light indeed introduced an imaging background as compared to the iDFM, the scattering intensity of the dominate colour after deduction of the MCB was still the highest in the corresponding MCB images, suggesting that it was not a simple superposition of the introduced background.

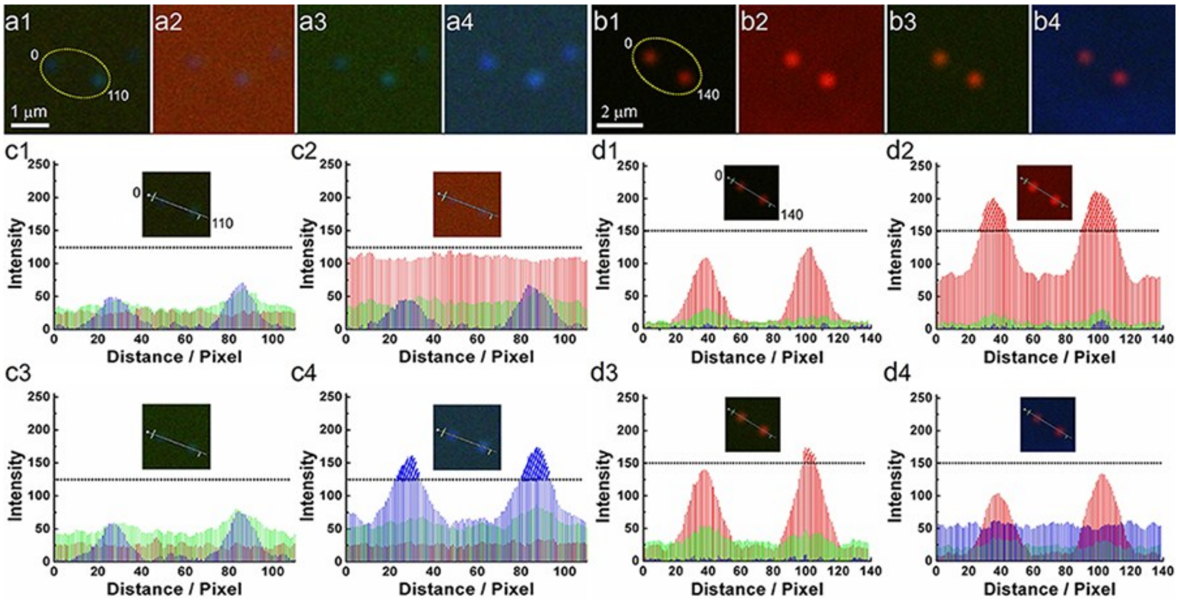

Figure S10 Enhanced scattering in RGB analysis. (a), (c) Scattering images and RGB line distribution of the blue nanoparticles in dark-field (a1, c1) and red, green and blue background imaging (a2-a4, c2-c4). (b), (d) Scattering images and RGB line distribution of the red nanoparticles in dark-field (b1, d1) and red, green and blue background imaging (b2-b4, d2-d4).

3.4. Scattering imaging of AgNPs with a filtered monochromatic light source.

The imaging with the refit condenser was different from the imaging illuminated by a monochromatic light source which was achieved by the filtration of the light source with a blue broadband filter (Fig. S11). In the monochromatic background imaging with the refit optical path, the scattering intensity of the nanoparticles scattering light in same colour was increased, however, the intensity was decreased by this filtering methods due to the reducing of light source intensity instead of enhancing the utilization efficiency (Fig. 12).

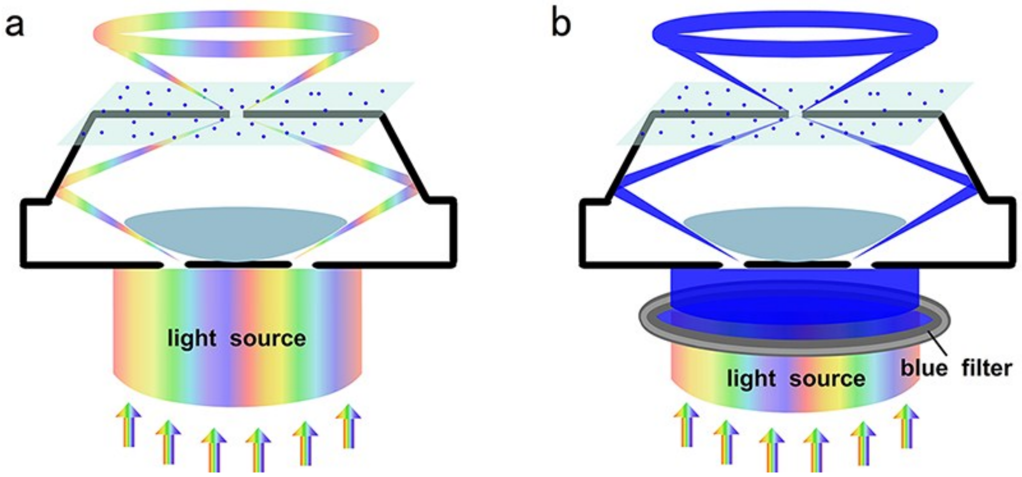

Figure S11 | Scattering imaging with monochromatic illumination by filtration of the light source. (a) Schematic diagram of the dark-field illumination. (b) Schematic diagram of the monochromatic illumination by filtration of the light source with a blue broadband filter.

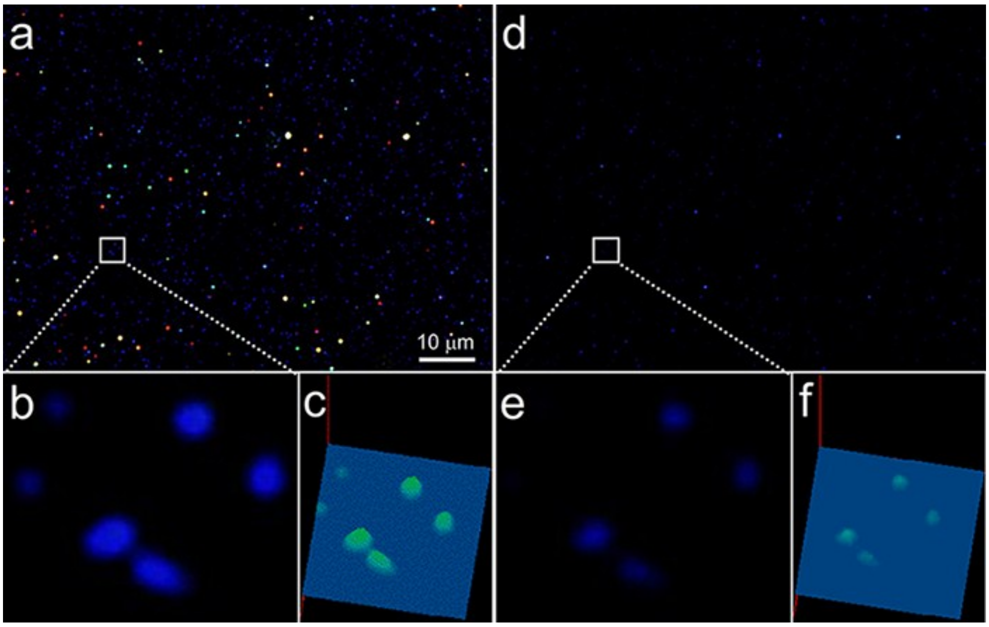

Figure S12 Scattering intensity loss with monochromatic illumination by filtration of the light source. (a) Dark-field image of AgNPs with the dark-field light source. (b) Enlarged image and (c) the three-dimensional model of the selected nanoparticles in the white square in a. (d) Scattering image of the same AgNPs with the monochromatic illumination obtained by the filtration of the dark-field light source with a blue broadband filter (center wavelength = 460 nm). (e) Enlarged image and (f) the three-dimensional model of the selected nanoparticles in the white square in d. The exposure time of these two imaging mode were same, and both were 300 ms.

3.5. Three-dimensional model and high-pass output of the scattering images.

The three-dimensional model of the scattering images was obtained by the "Surface Plot" function of Image-Pro Plus 6.0 Software. The principle of this function was grayscale conversion of the images and changing the image to a three-dimensional model with a variable angle. A typical rotation model was obtain by two procedure, elevation and rotation, and the process was showed in Fig. S13 in details.

The three-dimensional model of the blue nanoparticles under different imaging mode displayed the enhanced visibility under the blue background imaging. As shown in Fig. S14, the extended exposure time enhanced the scattering intensity of some nanoparticles and background simultaneously, but the noted two AgNPs had weak scattering intensities could only be distinguished clearly in the blue background image.

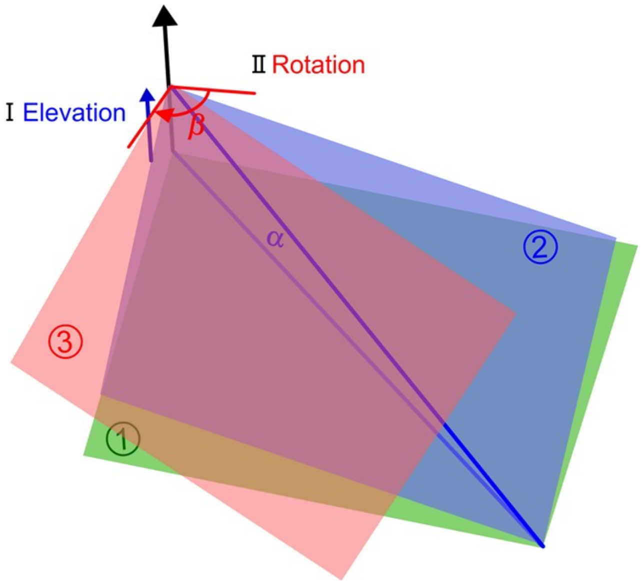

Figure S13 | Schematic diagram of the rotation process in the three-dimensional model of the scattering images. There are mainly two steps, elevation and rotation. Firstly, the image is elevated at a corner in the black arrow and its opposite corners is kept at the original plane (green rectangle), so there is a α degree angle formed between the image (blue rectangle) and the original plane (green rectangle). Then, the whole picture is rotated β degree clockwise and the last existing form (red rectangle) was obtained.

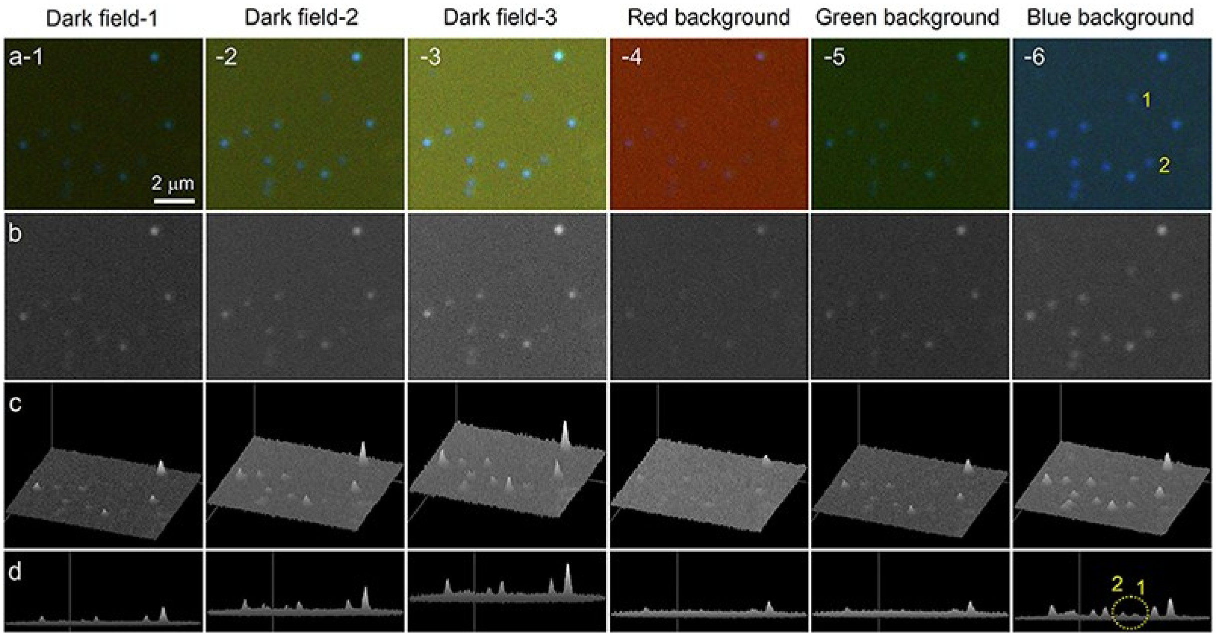

Figure S14 Three-dimensional model of the scattering images of the ~52.0 nm AgNPs. (a) Scattering images of the ~52.0 nm AgNPs and from a-1 to a-6 are the images in dark-field imaging with an exposure time of 300ms, 400ms, 500 ms, red, green and blue background imaging with an exposure time of 300ms. (b) The corresponding grayscale modes of the images in (a). (c) The rotated three-dimensional model of the images in (b). (d) Side view of the three-dimensional model and that the bump ups and downs easily display the visibility of the nanoparticles which can be clearly seen from the two noted nanoparticles.

4. PILC responsible for the visibility enhancement.

4.1 Scattering imaging of SiO_2 nanoparticles with the iCMF system.

From the scattering imaging and the RGB line distribution of SiO_2 nanoparticles in size of 180.5 nm, no obvious visibility enhancement was found (Fig. S15). The integral scattering intensity ((R+G+B)/3) of SiO_2 under different monochromatic imaging mode was almost the same as that under the dark-field imaging mode, and this was indeed different from that the imaging visibility of plasmonic nanoparticles was optimal under the same coloured monochromatic imaging mode (Fig. S16).

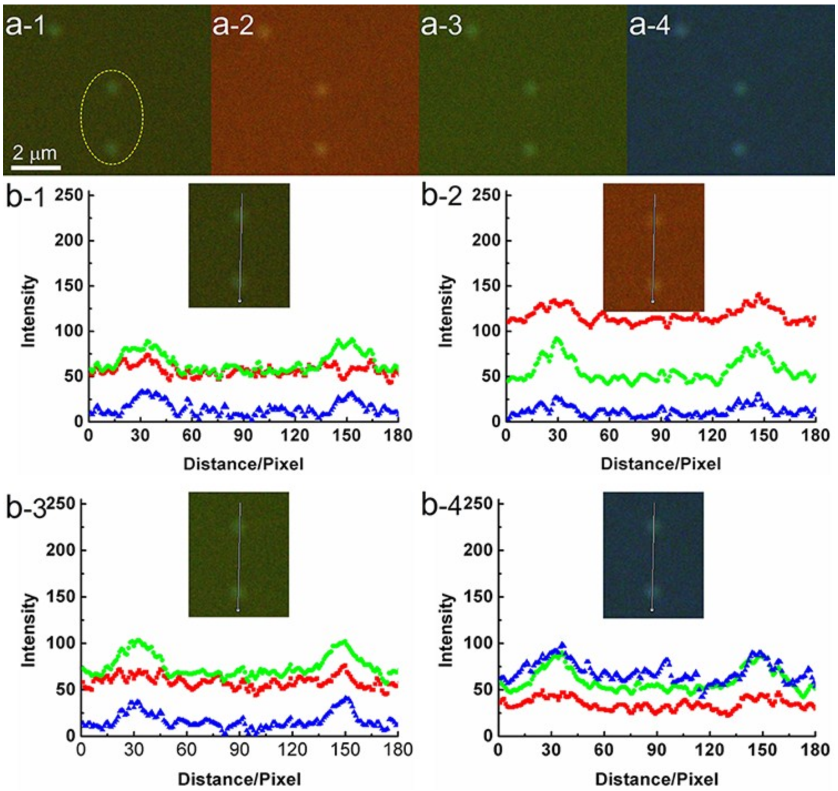

Figure S15 Scattering intensity in RGB analysis of SiO_2 nanoparticles. (a) Scattering images of the SiO_2 nanoparticles in size of 180.5 nm in dark-field and monochromatic background imaging, from the left to the right are red, green and blue. (b) Corresponding RGB line scan distribution of the SiO_2 nanoparticles in a).

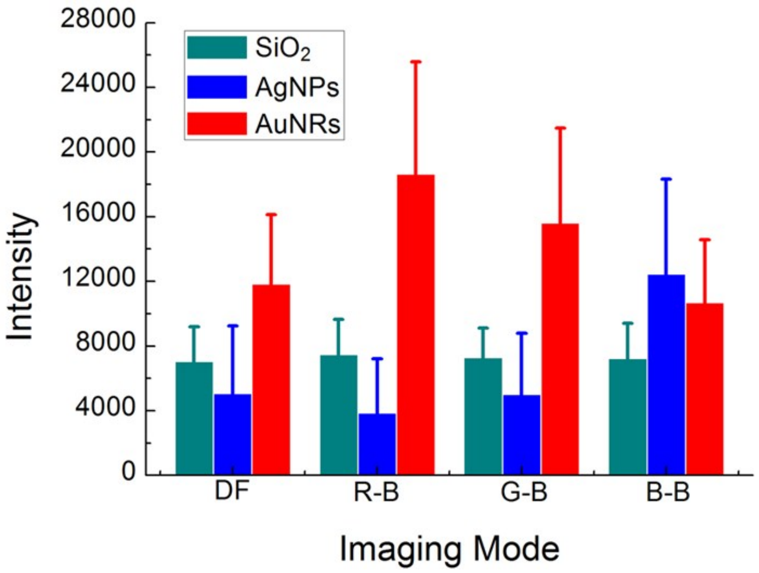

Figure S16 Scattering intensities of AgNPs, AuNRs and SiO_2 nanoparticles in different imaging modes. The scattering intensities of AgNPs, AuNRs and SiO_2 nanoparticles ((R+G+B)/3) in dark-field imaging and red, green and blue background imaging.

4.2 Finite-difference time-domain (FDTD) simulation.

Finite-difference time-domain (FDTD) simulation (FDTD Solutions 8.11.422, Lumerical Solutions, Inc., Canada) was utilized to calculate the extinction and scattering cross-section areas of the used plasmonic and nonplasmonic nanoparticles. During the calculation, a Gaussian light was used as the light source and launched into a box containing the nanoparticles. In the FDTD calculations, a nanoparticle was surrounded by a virtual boundary with an appropriate size. The nanoparticle and its surrounding medium inside the box were divided into 1 nm meshes. The size of the AgNPs was modeled as a perfect sphere (50 nm) and the individual AuNRs was modeled as a cylinder with two hemispherical end caps (75.4 nm in length, 38.5 nm in diameter). Both the scattering section area of 120 nm and 180 nm SiO_2 were calculated to compare the plasmonic and nonplasmonic nanoparticles. To further aware the light concentration of plasmonic nanoparticles, the electric field distribution of all the above nanoparticles were calculated and showed (z direction). All the nanoparticles were assumed to be embedded in water with a refractive index of 1.333.

From the calculated results showed in Fig. 3c-f, the AgNPs and AuNRs indeed had much larger extinction and scattering cross-section area than their physical cross-section area at their corresponding LSPR wavelength. Differently, the scattering cross-section areas of SiO_2 nanoparticles were much smaller than their physical cross section areas. Besides, around the SiO_2 nanoparticles, there were no local field enhancement, however, the field enhancement effect of AgNPs and AuNRs were significant. All the aforementioned results suggested that the light concentration effect was special property of plasmonic nanoparticles.

4.3 Wavelength-dependent scattering efficiency of AgNPs and AuNRs.

For spherical nanoparticles, the scattering efficiency could be given by Equation (S1)[8],

$$Q_{sca} = \frac{8}{3} x^4 \left| \frac{\varepsilon_i - \varepsilon_o}{\varepsilon_i + 2\varepsilon_o} \right|^2 \tag{S1}$$

Wherein $x = 2\pi a(\varepsilon_o)^{1/2}\lambda$, ε_i was wavelength-dependent dielectric constant of the metal particle and ε_o was dielectric constant of the surrounding medium.

For the ellipsoid, the scattering efficiency could be given by as Equation (S2)[9],

$$Q_{sca} = \frac{k^4}{6\pi s}\left(\frac{1}{3}|\alpha_1|^2 + \frac{1}{3}|\alpha_2|^2 + \frac{1}{3}|\alpha_3|^2\right) \quad \text{(S2)}$$

where $k = 2\pi(\varepsilon_o)^{1/2}/\lambda$, s was the cross-section area, α_1, α_2 and α_3 were the polarizabilities at the long axis direction and two perpendicular short axis. The α_n could be described by the following general formula as Equation (S3)[9],

$$\alpha_n = 4\pi abc \frac{\varepsilon_i - \varepsilon_o}{3\varepsilon_o + 3L_n(\varepsilon_i - \varepsilon_o)} \quad \text{(S3)}$$

where a, b and c was the semiaxis of the ellipsoid. As the transverse cross-section of nanorod was considered as a circle, so $c > a = b$. L_n was as Equation (S4)[9],

$$L_1 = \frac{abc}{2}\int_0^{\infty} \frac{dq}{(a^2 + q)f(q)} \quad \text{(S4)}$$

where $f(q) = [(q + a^2)(q + b^2)(q + c^2)]^{1/2}$. In L_2 and L_3, a was replaced by b and c, respectively.

5. Single nanoparticle spectra of AgNPs under different imaging modes.

The single nanoparticle scattering spectra of a blue and a red nanoparticles under the iDFM and iCFM illumination systems were measured and the results showed in Fig. 5b. It could be seen that the scattering spectra of the blue or red nanoparticles were kept in the nearly similar wavelength. Besides, the RGB value of the dominant colour and the total RGB value showed in Fig. S17 and S18 were consistent with the RGB analysis results is Fig. 3.

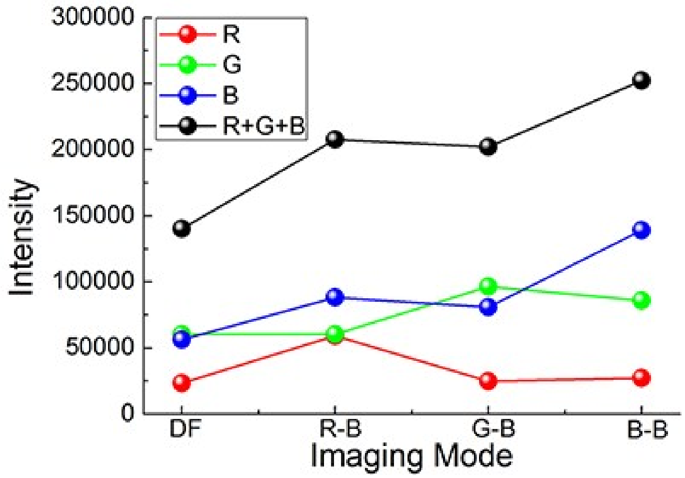

Figure S17 R, G, B and the RGB total value intensities of the blue nanoparticle in dark-field and monochromatic background scattering imaging. The image of the nanoparticle was in showed in Fig. 5b.

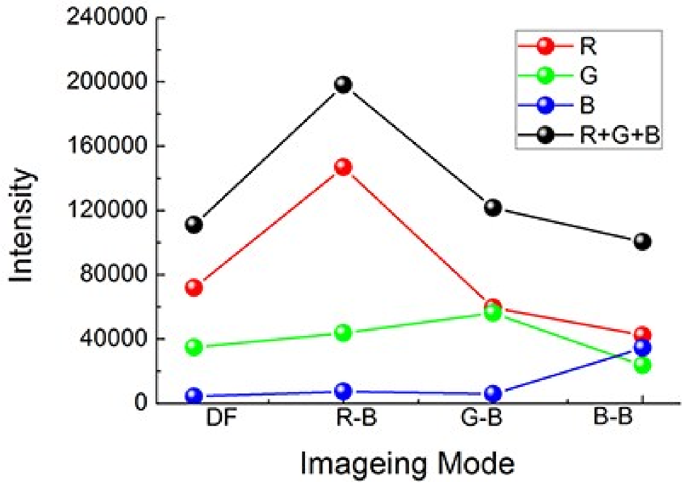

Figure S18 R, G, B and the RGB total value intensities of the red nanoparticle in dark-field and monochromatic background scattering imaging. The image of the nanoparticle was in showed in Fig. 5b.

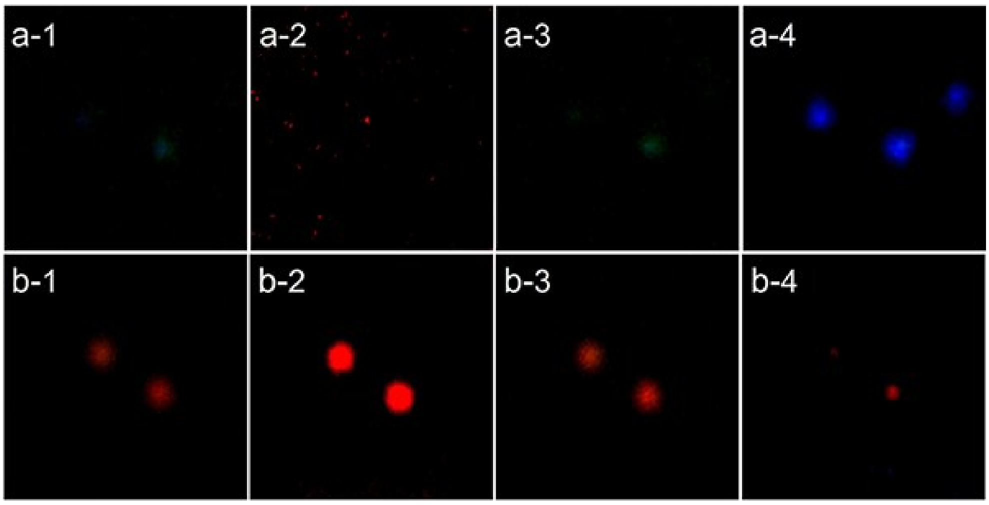

Figure 19 Background subtraction by simply contrast adjustment. a1-a4) The images after the background subtraction by contract adjustment corresponding to Fig. 5 d1. b1-b4) The images after the background subtraction by contract adjustment corresponding to Fig. 5 d3.

6. Hep-2 Cells-AgNPs scattering imaging with iCFM system.

The BSA protected AgNPs were prepared by a simple incubation of 1 mL of AgNPs which were mostly spheres and scattered blue light and 0.05 mL of 5% BSA for 30 min. The extinction spectra showed the narrow distribution of these spherical nanoparticles (Fig. S20a). To ensure the dispersed state of the AgNPs after the incubation with BSA, the BSA protected AgNPs was observed under the DFM with oil-immersed condenser and a 100× oil-immersed objective lens. The results showed in Fig. S20b revealed that the AgNPs were still dispersed and scattered mainly blue light.

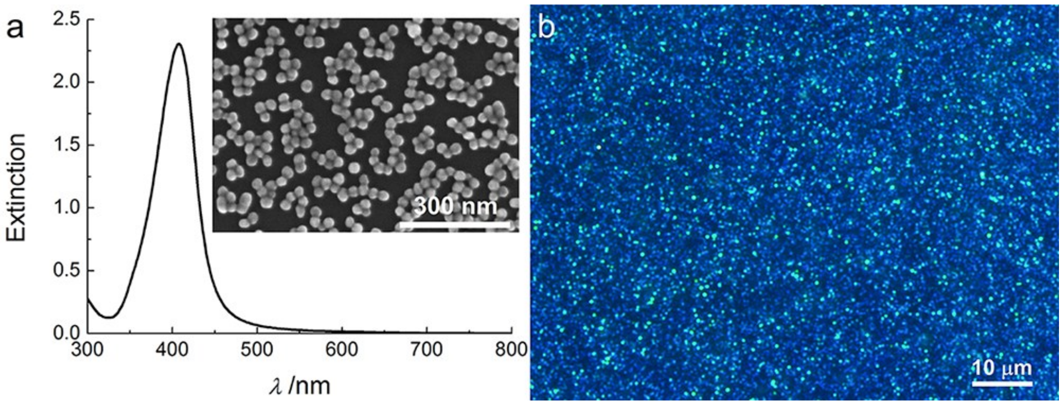

Figure S20 Characterization of the AgNPs used in the Hep-2 cancer cell imaging. (a) Extinction spectra of the silver nanoparticle and SEM images of the AgNPs. (b) Dark-field image of the AgNPs

after the coat of BSA.

The human laryngeal epithelial carcinoma cells (Hep-2 cancer cells) were plated onto cell culture dishes with a glass bottom (1 × 10^5 cells per dishes) and cultured in RPMI 1640 culture medium supplemented with 10% (v/v) fetal bovine serum (FBS) at 37°C with 5% CO_2. After incubation overnight, the cells were washed with PBS buffer solution. Then the AgNPs were incubated with the Hep-2 cancer cells in RPMI 1640 culture medium (2% FBS) for 30 min at 37°C with 5% CO_2. Afterwards, the cells were washed three times with PBS and fixed with 4% paraformaldehyde for 30 min at room temperature. Finally, the resulting solution was washed with PBS and deionized water twice, and then subjected to dark-field imaging and the monochromatic background imaging.

In imaging of the nanoparticles in the cancer cell, which was a complex environment, the nanoparticles might be located in different geometric level, so the depth of field was important in such case. Therefore, in imaging of BSA protected AgNPs, the used objective lens was a 100× oil immersion objective with a NA of 1.30, However, a 40× non-oil-immersed objective lens with a NA of 0.60 was used instead of the 100× oil-immersed objective lens in the imaging of the silver nanoparticle aggregates which had a different spot dimension with the dispersed AgNPs. In this way, the coexistence of these two existing state could be easily obtained.

The imaging results were showed in Fig. S21 and the protection of the BSA was obvious from the scattering imaging. Among these imaging modes with the dry type condenser, it was also that the imaging with the corresponding monochromatic background imaging displayed the optimal quality and visibility. The RGB line distribution analysis of a single blue nanoparticle and a single red nanoparticles also revealed the same results (Fig. S22).

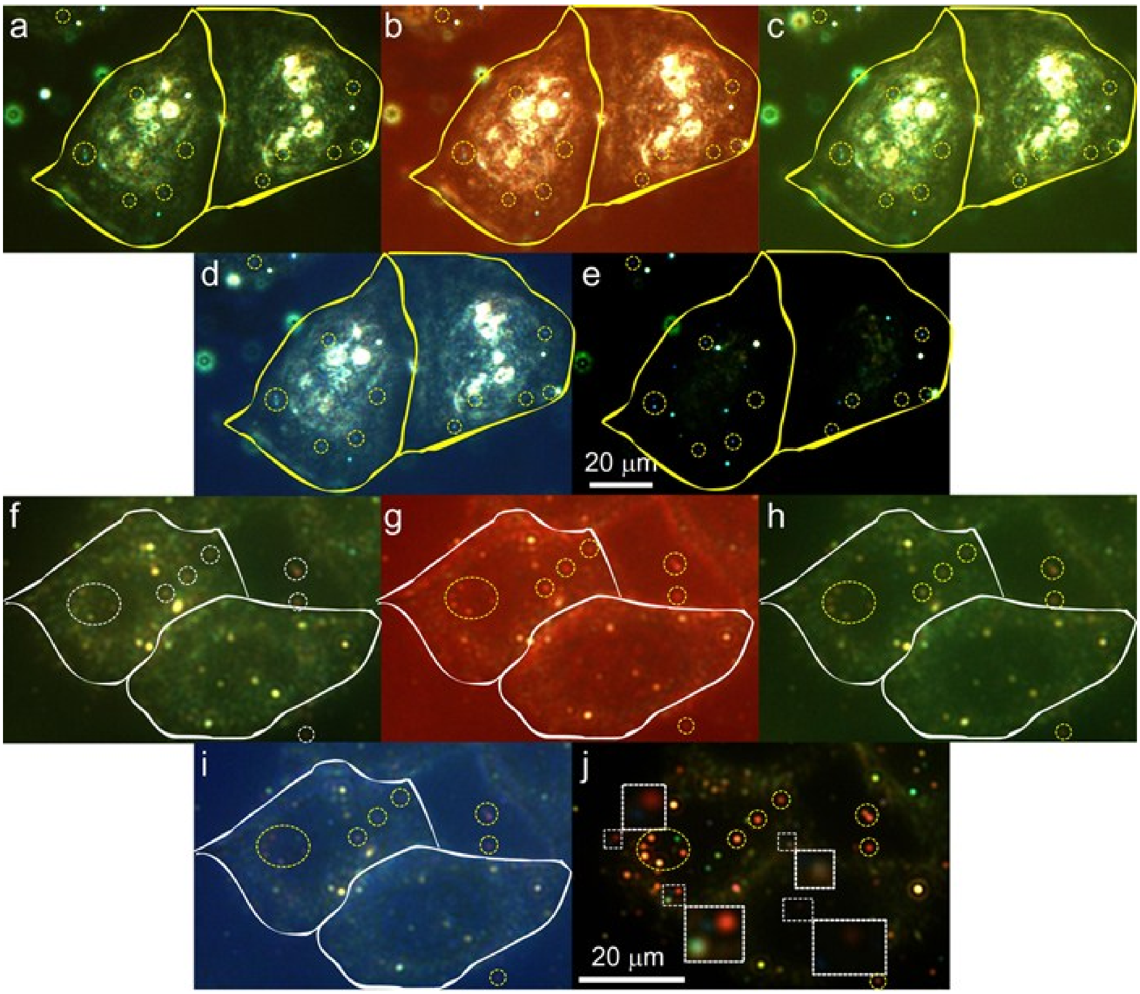

Figure S21 | Scattering images of the AgNPs protected by BSA or not after incubated with Hep-2 cancer cells. (a)-(d) Scattering images of the AgNPs protected by BSA after incubation with Hep-2 cancer cells in dark-field imaging, red, green, blue background imaging with iCFM system. (e) Scattering images obtained with the oil-immersed condenser. (f)-(i) Scattering images of the unprotected AgNPs after incubation with Hep-2 cancer cells in dark-field imaging, red, green, blue background imaging with refit U-DCD condenser. (j) Scattering images obtained with the oil-immersed condenser, and all the f-j were imaged with a 40× non-oil-immersed objective lens with a NA value of 0.60. The enlarged images in squares in j showed the obvious scattering intensity difference between the blue nanoparticles and the aggregates.

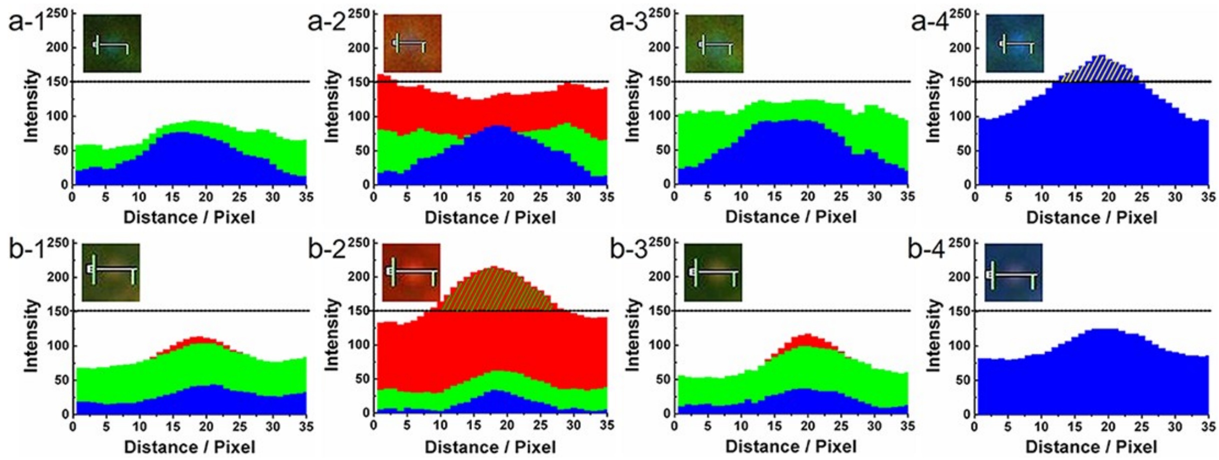

Figure S22 | RGB line distribution of the silver nanoparticle and aggregates in the scattering imaging. a) RGB line distribution of the AgNP noted as no. 3 in fig. 6a. b) RGB line distribution of the aggregated AgNP noted as no. 5 in fig. 6c. In all these RGB distribution, from the bottom to the top, red, green and blue colour are distributed sequentially.

SUPPLEMENTARY REFERENCES

1. Steinigeweg, D. & Schlucker, S. Monodispersity and size control in the synthesis of 20-100 nm quasi-spherical silver nanoparticles by citrate and ascorbic acid reduction in glycerol-water mixtures. *Chem. Commun.* **48**, 8682-8684, (2012).
2. Ni, W., Kou, X., Yang, Z. & Wang, J. Tailoring Longitudinal Surface Plasmon Wavelengths, Scattering and Absorption Cross Sections of Gold Nanorods. *ACS Nano* **2**, 677-686, (2008).
3. Zhang, Z. *et al.* Mesoporous Silica-Coated Gold Nanorods as a Light-Mediated Multifunctional Theranostic Platform for Cancer Treatment. *Adv. Mater.* **24**, 1418-1423, (2012).
4. Stöber, W., Fink, A. & Bohn, E. Controlled growth of monodisperse silica spheres in the micron size range. *J. Colloid Interf. Sci.* **26**, 62-69, (1968).
5. Wong, Y. J. *et al.* Revisiting the Stöber Method: Inhomogeneity in Silica Shells. *J. Am. Chem. Soc.* **133**, 11422-11425, (2011).
6. Liu, Y. & Huang, C. Z. Real-Time Dark-Field Scattering Microscopic Monitoring of the in Situ Growth of Single Ag@Hg Nanoalloys. *ACS Nano* **7**, 11026-11034, (2013).
7. Zhou, W., Bovik, A. C., Sheikh, H. R. & Simoncelli, E. P. Image quality assessment: from error visibility to structural similarity. *IEEE Trans. Image Process.* **13**, 600-612, (2004).
8. Kelly, K. L., Coronado, E., Zhao, L. L. & Schatz, G. C. The Optical Properties of Metal

Nanoparticles: The Influence of Size, Shape, and Dielectric Environment. *J. Phys. Chem. B* **107**, 668-677, (2003).

9. Bohren, C. F. & Huffman, D. R. *Absorption and Scattering of Light by Small Particles*, Wiley-VCH Verlag GmbH, (1998).
